# Polygenic scores for obstructive sleep apnoea reveal pathways contributing to cardiovascular disease

**DOI:** 10.1016/j.ebiom.2025.105790

**Published:** 2025-06-04

**Authors:** Nuzulul Kurniansyah, Satu J. Strausz, Geetha Chittoor, Shreyash Gupta, Anne E. Justice, Yana Hrytsenko, Brendan T. Keenan, Brian E. Cade, Brian W. Spitzer, Heming Wang, Jennifer Huffman, Matthew R. Moll, Bernhard Haring, Su Yon Jung, Laura M. Raffield, Robert Kaplan, Jerome I. Rotter, Stephen S. Rich, Sina A. Gharib, Traci M. Bartz, Peter Y. Liu, Han Chen, Myriam Fornage, Lifang Hou, Daniel Levy, Alanna C. Morrison, Heather M. Ochs-Balcom, Bruce M. Psaty, Peter W.F. Wilson, Kelly Cho, Allan I. Pack, Hanna M. Ollila, Susan Redline, Daniel J. Gottlieb, Tamar Sofer, FinnGen, The Trans-Omics in Precision Medicine Consortium, The VA Million Veteran Program

**Affiliations:** a VA Boston Healthcare System, Boston, MA, USA; b Division of Sleep and Circadian Disorders, Department of Medicine, Brigham and Women's Hospital, Boston, MA, USA; c Department of Bioinformatics, Boston University, Boston, MA, USA; d Institute for Molecular Medicine Finland, Helsinki Institute of Life Science, University of Helsinki, Helsinki, Finland; e Department of Genetics, Stanford University School of Medicine, Stanford, CA, USA; f Department of Oral and Maxillofacial Diseases, Helsinki University Hospital, Helsinki, Finland; g Department of Cleft Palate and Craniofacial Center, University of Helsinki and Helsinki University Hospital, Helsinki, Finland; h Orthodontics, Department of Oral and Maxillofacial Diseases, Clinicum, Faculty of Medicine, University of Helsinki, Helsinki, Finland; i Department of Population Health Sciences, Geisinger Health System, Danville, PA, USA; j Department of Medicine, Harvard Medical School, Boston, MA, USA; k CardioVascular Institute (CVI), Beth Israel Deaconess Medical Center, Boston, MA, USA; l Division of Sleep Medicine, Department of Medicine, University of Pennsylvania, Philadelphia, PA, USA; m Massachusetts Veterans Epidemiology Research and Information Center, Veterans Affairs Boston Healthcare System, Boston, MA, USA; n VA Palo Alto Health Care System, Palo Alto, CA, USA; o Palo Alto Veterans Institute for Research, Palo Alto, CA, USA; p Channing Division of Network Medicine, Brigham and Women's Hospital, Boston, MA, USA; q Division of Pulmonary and Critical Care Medicine, Brigham and Women's Hospital, Boston, MA, USA; r Section on Pulmonary, Critical Care, Sleep, and Allergy, Veterans Affairs Boston Healthcare System, West Roxbury, MA, USA; s Department of Epidemiology and Population Health, Albert Einstein College of Medicine, Bronx, NY, USA; t Department of Medicine III, Saarland University, Homburg, Germany; u Department of Epidemiology, Fielding School of Public Health, University of California, Los Angeles, CA, USA; v Translational Sciences Section, School of Nursing, Jonsson Comprehensive Cancer Center, University of California, Los Angeles, CA, USA; w Department of Genetics, University of North Carolina at Chapel Hill, Chapel Hill, NC, USA; x Division of Public Health Sciences, Fred Hutchinson Cancer Center, Seattle, WA, USA; y The Institute for Translational Genomics and Population Sciences, Department of Pediatrics, The Lundquist Institute for Biomedical Innovation at Harbor-UCLA Medical Center, Torrance, CA, USA; z Department of Genome Sciences, University of Virginia School of Medicine, Charlottesville, VA, USA; aa Cardiovascular Health Research Unit, Department of Medicine, University of Washington, Seattle, WA, USA; ab Division of Pulmonary, Critical Care and Sleep Medicine, Computational Medicine Core, Center for Lung Biology, University of Washington, Seattle, WA, USA; ac Department of Biostatistics, University of Washington, Seattle, WA, USA; ad Division of Genetics, Lundquist Institute at Harbor-UCLA Medical Center, Torrance, CA, USA; ae Human Genetics Center, Department of Epidemiology, School of Public Health, The University of Texas Health Science Center at Houston, Houston, TX, USA; af Brown Foundation Institute of Molecular Medicine, McGovern Medical School, University of Texas Health Science Center at Houston, Houston, TX, USA; ag Department of Preventive Medicine, Feinberg School of Medicine, Northwestern University, Chicago, IL, USA; ah The Population Sciences Branch of the National Heart, Lung and Blood Institute, Bethesda, MD, USA; ai The Framingham Heart Study, Framingham, MA, USA; aj Department of Epidemiology and Environmental Health, University of Buffalo, Buffalo, NY, USA; ak Department of Epidemiology, University of Washington, Seattle, WA, USA; al Department of Medicine, University of Washington, Seattle, WA, USA; am Health Systems and Population Health, University of Washington, Seattle, WA, USA; an Atlanta VA Healthcare System, Decatur, GA, USA; ao Emory Clinical Cardiovascular Research Institute, Atlanta, GA, USA; ap Division of Aging, Brigham and Women's Hospital, Department of Medicine, Boston, MA, USA; aq Circadian Sleep Institute, Perelman School of Medicine, University of Pennsylvania, Philadelphia, PA, USA; ar Division of Sleep Medicine, Department of Medicine, Perelman School of Medicine, University of Pennsylvania, Philadelphia, PA, USA; as Department of Anesthesia, Critical Care and Pain Medicine, Massachusetts General Hospital and Harvard Medical School, Boston, MA, USA; at Center for Genomic Medicine, Massachusetts General Hospital and Harvard Medical School, Boston, MA, USA; au Broad Institute of Harvard and MIT, Cambridge, MA, USA; av Department of Biostatistics, Harvard T.H. Chan School of Public Health, Boston, MA, USA

**Keywords:** Genetically determined OSA, Sex differences, Body fat distribution, Diverse populations

## Abstract

**Background:**

Obstructive sleep apnoea (OSA) is a common chronic condition, with obesity its strongest risk factor. Polygenic scores (PGSs) summarise the genetic liability to phenotype and can provide insights into relationships between phenotypes. Recently, large datasets that include genetic data and OSA status became available, providing an opportunity to utilise PGS approaches to study the genetic relationship between OSA and other phenotypes, while differentiating OSA-specific from obesity-specific genetic factors.

**Methods:**

Using race/ethnic diverse samples from over 1.2 million individuals from the Million Veteran Program, FinnGen, TOPMed, All of Us (AoU), Geisinger's MyCode, MGB Biobank, and the Human Phenotype Project, we developed and assessed PGSs for OSA, both without (BMIunadjOSA-PGS) and with adjustment for the genetic contributions of BMI (BMIadjOSA-PGS).

**Findings:**

Adjusted odds ratios (ORs) for OSA per 1 standard deviation of the PGSs ranged from 1.38 to 2.75. The associations of BMIadjOSA- and BMIunadjOSA-PGSs with CVD outcomes in AoU shared both common and distinct patterns. Only BMIunadjOSA-PGS was associated with type 2 diabetes, heart failure, and coronary artery disease, while both BMIadjOSA- and BMIunadjOSA-PGSs were associated with hypertension and stroke. Sex stratified analyses revealed that BMIadjOSA-PGS association with hypertension was driven by females (OR = 1.1, p-value = 0.002, OR = 1.01 p-value = 0.2 in males). OSA PGSs were also associated with body fat measures with some sex-specific associations.

**Interpretation:**

Distinct components of OSA genetic risk are related and independent of obesity. Sex-specific associations with body fat distribution measures may explain differing OSA risks and associations with cardiometabolic morbidities between sexes.

**Funding:**

R01AG080598.

**Research in context**

**Evidence before this study**

Polygenic scores (PGSs), summarizing the genetic disposition to a phenotype or trait, have been shown to be effective in characterizing the genetic mechanisms underlying traits and for studying genetically-determined relationships between traits. However, few manuscripts have reported the development of PGS for obstructive sleep apnoea (OSA), limiting the application of genetic analysis techniques that use PGSs to study OSA. Further, these previously-developed OSA PGSs were mostly based on homogenous populations of European ancestry, or a limited number of genetic variants, and did not comprehensively adjust for the impact of body mass index (BMI), and did not conduct sex-stratified analyses on genetic associations with OSA.

**Added value of this study**

Compared to previous studies, the current study used more diverse populations, larger sample sizes, and modern, powerful, PGS methods to develop PGSs for OSA. Further, we developed two OSA PGSs: one summarizing all genetic effects on OSA, and one accounting for BMI-related effects, capturing genetic effects on OSA that are independent of BMI. We estimated the associations of these OSA PGSs with OSA in multiple independent studies, and further performed analyses stratified by sex, age, and race/ethnicity strata. These analyses affirm that the new OSA PGSs have stronger associations with OSA compared to those previously reported, and that the PGS associations are similar in males and females and across age strata. Moreover, we demonstrate that PGSs that capture BMI-independent genetic effects on OSA have stronger associations with OSA in BMI-adjusted analyses than do PGSs developed without accounting for BMI. We also analysed OSA PGS associations with other sleep measures, with cardiovascular, pulmonary, and metabolic outcomes, and with measures of body fat distribution. These analyses indicate an association of OSA PGS with the proportion of visceral adipose tissue android fat, and an inverse association with the proportion of gynoid fat. The analyses also identified certain outcomes where the associations with the BMI-adjusted and BMI-unadjusted OSA PGSs were similar, including hypertension, stroke and atrial fibrillation, and other outcomes where the associations with the two PGSs were very different, including type 2 diabetes, heart failure, and chronic obstructive pulmonary disease.

**Implications of all the available evidence**

The present study greatly expands our understanding of the genetic determinants of OSA. The available evidence suggests that OSA PGSs may be useful for identifying individuals at increased risk of developing OSA, independent of known risk factors such as age, sex and BMI. Moreover, genetically-determined OSA is associated with increased risk of a range cardiovascular, metabolic, and pulmonary outcomes. The present study demonstrates that OSA PGSs are associated with more deleterious body fat distribution measures. However, BMI-unadjusted and BMI-adjusted OSA PGSs differ in their association with a number of health conditions, suggesting differences in the mechanisms underlying the association of OSA with specific cardiopulmonary and metabolic comorbidities.

## Introduction

Obstructive sleep apnoea (OSA) is a common disorder with an estimated prevalence of 17% (women) and 34% (men) in middle aged U.S. adults.^1^ OSA is associated with increased risk of cardiometabolic diseases, such as type 2 diabetes and hypertension, as well as increased rates of cardiovascular diseases (CVDs) including stroke, ischaemic heart disease, and others.^2^, ^3^, ^4^ Despite recognition of the high prevalence of OSA, it remains underdiagnosed,^5^^,^^6^ with likely lower rates of diagnosis in women and in individuals who are less symptomatic (i.e., have fewer comorbidities such as hypertension or diabetes).^7^ Major OSA risk factors include obesity, male sex, and older age.^8^

Polygenic scores (PGSs) summarise genetic contributions to a trait as linear combinations of many trait-associated genetic alleles. PGSs are being increasingly developed and are being applied to assess genetic relationships between phenotypes^9^, ^10^, ^11^ and identify changes in genetic contributions by age, lifestyle, and environment.^12^, ^13^, ^14^, ^15^ Importantly, PGSs are being studied and sometimes translated into clinical use, such as for risk prediction, stratification and classification into risk groups, and for screening.^16^^,^^17^ PGSs have also been used for characterizing etiologic pathways.^18^^,^^19^ PGSs for OSA may help advance the understanding of OSA given the heterogeneity of OSA phenotypes, its complex associations with other chronic diseases, and the incomplete understanding of its underlying mechanisms.^20^ Ultimately, OSA PGSs may lead to improved risk stratification, screening, and treatment that targets underlying disease mechanisms.

To develop and assess OSA PGSs, we used data from racially and ethnically diverse samples from multiple biobanks and cohort studies, including the Million Veteran Program (MVP,^21^), FinnGen,^22^ TOPMed, All of Us, Geisinger's MyCode, Mass General Brigham (MGB) Biobank, and the Human Phenotype Project (HPP). Given the known sex differences in OSA,^23^ and the strong effect of obesity on OSA risk, we use sex stratified summary statistics from genome-wide association studies (GWAS) of OSA without BMI adjustment from three of these cohorts (the MVP, FinnGen, and Han Chinese population GWAS) and after adjusting for BMI for two cohorts (MVP and FinnGen) to develop predictive OSA PGSs. We then constructed the PGSs in other studies. Earlier epidemiological and genetic work has demonstrated that BMI is the strongest causal risk factor for OSA.^24^ Therefore, it is critical to understand how BMI contributes to comorbidities and relationships between OSA and CVD. Ultimately, this potentially allows us to understand BMI-dependent and BMI-independent cardiovascular consequences of OSA. To achieve these aims, we assessed the associations of the PGSs with OSA, OSA-related measures, other sleep phenotypes, and cardio-pulmonary, metabolic and kidney disease outcomes, in multiple independents studies including individuals representing diverse populations.

## Methods

**Fig. 1:**
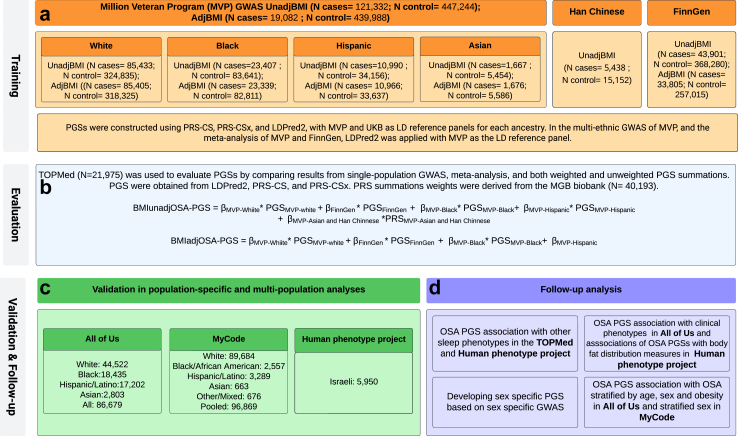
**OSA PGS development and assessment**. Development and assessment of OSA PGSs. The steps are composed of (a) PGS training using GWAS summary statistics, reference panels, and a separate population for computing PGS summation weights; (b) evaluation step used to select PGS out of multiple candidates; (c) validation of associations with OSA in new independent datasets; and (d) follow up analyses addressing OSA PGS associations with OSA within various strata, associations with related sleep phenotypes, comorbidities, and sequelae of OSA. Analyses in step (d) were in datasets from previous steps. OSA, obstructive sleep apnoea; PGS, polygenic score; GWAS, genome-wide association study.

Fig. 1 provides an overview of the study. In brief, we used GWAS summary statistics from multiple published GWAS of OSA using three modern methods, LDPred2,^25^ PRS-CS,^26^ and PRS-CSx,^27^ that have been shown to be very successful for polygenic, complex, traits. We developed PGSs after either meta-analysing GWAS summary statistics across independent cohorts, or using a PGS combination approach, as a weighted sum. We then evaluated PGSs in association with OSA, and performed additional analyses where we studied PGS associations with other phenotypes.

### GWAS summary statistics

We developed PGS based on summary statistics from MVP,^21^ from the FinnGen study,^22^ and from a GWAS of OSA in Han Chinese.^28^ Table 1 provides information about the summary statistics.

**Table 1:** GWAS summary statistics used for OSA PGS development.

GWAS	Reference	Sex group	BMI adjustment	Sex-combined sample size	Study population
MVP	PMID 36989840	Combined, and sex-stratified	Unadjusted	Cases: 121,332 Controls: 447,244	91% male, 72% White, 19% Black, 8% Hispanic, 1.2% Asian.
MVP	PMID 36989840	Combined, and sex-stratified	Adjusted	Cases: 119,082 Controls: 439,988	91% male, 72% White, 19% Black, 8% Hispanic, 1.2% Asian.
FinnGen release 10	PMID 36653562	Combined, and sex-stratified	Unadjusted	Cases: 43,901; Controls: 368,280	Finnish Europeans
FinnGen release 10	PMID 36653562	Combined, and sex-stratified	Adjusted	Cases: 33,805; Controls: 257,015	Finnish Europeans
Han Chinese	PMID 35819321	Combined	Unadjusted	Cases: 5438; Controls: 15,152	Han Chinese

MVP study population was defined by “HARE group”: groups defined by a harmonised genetic ancestry and self-reported race/ethnicity algorithm^29^ designed to create genetically similar groups, with group definitions relying on self-reported race/ethnicity of the majority of group members.

### GWAS of OSA in FinnGen

The FinnGen study is a large-scale genomics initiative that has analysed over 500,000 Finnish biobank samples and correlated genetic variation with health data to understand disease mechanisms and predispositions. The project is a collaboration between research organizations and biobanks within Finland and international industry partners.

The project aims to identify the impact of genetic and environmental factors on various diseases while enhancing understanding of the progression and biological mechanisms underlying these conditions.^30^ Genome-wide association testing for OSA was performed utilizing Regenie 2.2.4^31^ through the FinnGen Regenie pipelines (https://github.com/FINNGEN/regenie-pipelines). The analysis was adjusted for the current age or age at death, sex, genotyping chip, genetic relatedness, and the first 10 genetic PCs. The analysis was also conducted adjusting for BMI, and it was repeated for both sexes separately.

### Creating an MVP LD reference panel for PGS development

We used three popular methods to develop PGSs: LDPred2, PRS-CS, and PRS-CSx, all requiring the use of reference panels—correlation matrices tabulating the linkage disequilibrium (LD; correlation) between single nucleotide polymorphisms (SNPs) used for PGS preparation. We constructed such matrices based on the MVP dataset, using unrelated individuals only (overall N = 567,748).

We focused on HapMap SNPs, and further required, for each LD reference panel representing a specific population subset, MAF of at least 0.01, and imputation quality ≥0.8. We excluded SNPs from the major histocompatibility complex (MHC) region (chr6: 26–34 Mb; grch37), We first used the R package bigsnpr (V1.12.) to create correlation matrices based on each of MVP HARE groups, and based on the combined sample. For the European HARE group, we subset the dataset to 100 K unrelated individuals, to speed up computing time. Because other HARE groups had smaller sample sizes, we used all unrelated individuals associated with the group (N African: 106,445, N Hispanic: 44,714, N Asian: 7149). To create the multi-population correlation matrix, we sampled 100 K individuals at random from the unrelated set of MVP individuals. Random sampling ensured that the proportions of individuals from each HARE group was maintained from the complete dataset. LDPred2 requires correlation matrices based on entire chromosomes. Thus, we used function snp_cor from bigsnpr to compute these matrices for each chromosome, HARE group, and the multi-population dataset.

PRS-CS and PRS-CSx required splitting each chromosome to smaller LD blocks, typically of hundreds of SNPs^26^ due to computational limitations. Thus, we applied the snp_ldsplit function from the form bigsnpr package to split the correlation matrices from each chromosome, in each HARE group dataset, to LD blocks, varying the r^2^ parameters and minimum and maximum block sizes as needed (following the bigsnpr tutorial). This function was not able to identify independent LD blocks based on the multi-population dataset based on a range of parameters (we considered r^2^ up to 0.3), likely due to admixture, so we did not apply PRS-CS on the multi-population GWAS summary statistics and only used it based on individual HARE group summary statistics.

For PRS-CS and PRS-CSx, we outputted the SNPs in each LD block, used plink v.1.9 to create correlation matrices, and next combined them by chromosome.

### PGS development

We developed PGSs using LDPred2, PRS-CS, and PRS-CSx. The latter method is a multi-PGS combination method that relies on summary statistics from GWAS performed in populations of distinct ancestral make-up and develops PGS matching each of the ancestries, to be later combined as a weighted (or unweighted) sum. We used the reference panels developed in MVP as well as the reference panels provided with the PRS-CS and PRS-CSx software, which are based on subpopulations of the UKB program, and develop PGS for OSA based on BMI-adjusted and BMI-unadjusted analyses. We also developed PGSs using PRS-CS and LDPred2 and combined them as sums of PGSs. We considered the following PGS constructions:

1. **Only based on MVP, HARE group specific GWAS:** using LDPred2 based on MVP HARE group reference panels; using PRS-CS and PRS-CSx based on MVP HARE group LD reference panels; using PRS-CS and PRS-CSx based on UKB LD reference panels. The difference between PRS-CS and PRS-CSx is that PRS-CSx constructs the HARE groups (or ancestry)-specific PGS while borrowing information from other groups while PRS-CS does not.
2. **Only using FinnGen:** using LDPred2 and PRS-CS based on MVP and UKBB European reference panels.
3. **Based on similar ancestry meta-analysis of MVP, FinnGen, and Han Chinese OSA GWAS summary statistics.** Here, we meta-analysed MVP Asian + Han Chinese OSA GWAS (only BMI unadjusted due to data availability), and meta-analysed MVP White HARE group with FinnGen Finish Europeans. We then used the same approaches as in bullet 1 above.
4. **PGS based on multi-population meta-analysis:** using the meta-analysis of summary statistics of MVP only, and similarly using meta-analysis of all MVP, FinnGen, and Han Chinse GWAS, with LDPred2 and the multi-population MVP reference panel.

Ancestry/population-specific PGSs developed as described in bullet points 1–3 above were combined together (as described below) as weighted or unweighted sums.

We also developed PGSs based on sex-specific GWAS summary statistics. Because the sample size of female participants is small in MVP, especially of non-White participants, for female sex PGSs we developed MVP-only PGS using the multi-ethnic female GWAS results with LDPred2, and a European population-based PGS using the White MVP female GWAS summary statistics meta-analysed with the FinnGen female GWAS summary statistics. For male-only PGS, we used the same methods as described above for the sex-combined PGSs.

### MGB biobank

The Mass General Brigham (MGB) biobank is a biorepository of consented patient samples at MGB (Supplementary Note 2). We extracted data from MGB biobank in November 2021. OSA status was extracted from the field “Obstructive Sleep Apnoea OSA”. We used the dataset to derive PGS combination weights. In brief, we used^1^ PRS-CSx PGSs as well as, separately,^2^ single population PGSs (MVP HARE groups only and combined with FinnGen and Han Chinese OSA GWAS) developed using LDPred2 or (separately) PRS-CS, in logistic regression of OSA over the set of PGSs. The effect estimates of the PGSs are the weights used later to create a sum of PGSs, e.g.: w_1_xPGS_1_ + w_2_xPGS_2_ + w_3_xPGS_3_. Analyses in MGB Biobank were adjusted for current age, sex, self-reported race/ethnicity, genotyping batch, and BMI (linear and squared terms), with BMI being the median BMI in the health records for each person. We performed both BMI-unadjusted and BMI-adjusted analyses, where the adjustment in MGB matched the BMI adjustment used in the original GWAS from which the relevant PGSs were constructed by.

### Deriving a single set of PGS variant weights from a multiple PGS combination

To facilitate replication of PGS association analyses as well as general use of PGS in external studies, we here demonstrate how we derive a new PGS, that accounts for the weighted (or unweighted) summation of multiple, standardised, PGSs. The formula for the k th PGS is:

$$\frac{1}{2p_{k}}\sum_{i=1}^{p_{k}}g_{i}\beta_{ki}$$

where note that is standardised according to the number of potential alleles (2 times the number of variants). Suppose that we computed the mean and standard deviation of the PGSs in the evaluation dataset (here, TOPMed), and they are given by $\mu_{k},\sigma_{k}$ for the k PGS. Noting that the number of variants can change between PGSs, we denote by $p_{k}$ the number of variants in the k th PGS. When summing K PGSs with weights, $w_{1},\ldots ,w_{K}$ the expression is:

$$\text{wPGSsum}=w_{1}\frac{\left(\frac{1}{2p_{1}}\sum_{i=1}^{p_{1}}g_{i}\beta_{1i}-\mu_{1}\right)}{\sigma_{1}}+\ldots +w_{K}\frac{\left(\frac{1}{2p_{k}}\sum_{i=1}^{p_{k}}g_{i}\beta_{Ki}-\mu_{K}\right)}{\sigma_{K}}$$

This can alternatively be written as a single PGS formula after some rearrangement of terms, and harmonizing all variants to be considered as being a single set of p variants, with a variant potentially having a β weight of zero in any specific PGS, then:

$$\text{wPGSsum}=\sum_{i=1}^{p}g_{i}\left(\frac{w_{1}}{{2p_{1}\sigma }_{1}}\beta_{1i}+\ldots +\frac{w_{K}}{{2p_{k}\sigma }_{K}}\beta_{Ki}\right)-\left(\frac{w_{1}\mu_{1}}{2p_{1}\sigma_{1}}+\ldots +\frac{w_{K}\mu_{K}}{{2p_{k}\sigma }_{K}}\right)$$

Therefore, we can compute wPGSsum as a single PGS, with the β weight for each variant being obtained as a weighted sum of the β weights of the individual PGSs. In other words, we can multiply the variant β weights in the k PGS by $w_{k}/\left(2p_{k}\sigma_{k}\right)$, with $w_{k}$ being the weight from the PGS summation, and $\sigma_{k}$ being the PGS standard deviation in the TOPMed dataset.

### The TOPMed dataset

The TOPMed dataset provides aggregated whole genome sequencing (WGS) across multiple studies. We used WGS data to construct and assess PGS associations using several studies with available OSA phenotypes. Some studies performed at-home, over-night sleep studies (ARIC, CARDIA, CHS, and FHS via the Sleep Heart Heath Study,^32^ CFS,^33^ HCHS/SOL,^34^ JHS,^35^ MESA^36^), and only one study used a questionnaire-based OSA status (COPDGene). Detailed descriptions of these studies are provided in Supplementary Note 3.

#### OSA phenotype used in PGS assessment

Supplementary Table S1 describe the OSA assessment across these studies. In brief, we defined OSA in studies that used over-night measurements using the apnoea-hypopnea index (AHI) or the respiratory event index (REI) as defined by each study, with AHI/REI ≥5 (mild-to-severe OSA) and with AHI/REI ≥15 (moderate-to-severe OSA). Because COPDGene used self-reported doctor diagnosed OSA, while other TOPMed studies used a quantitative index, we compared using OSA definition, for studies using an index, as either mild-to-severe OSA versus no OSA, or using moderate-to-severe OSA versus no or mild OSA. To focus the potential analyses, this comparison was performed using the full available TOPMed OSA dataset and the multi-ethnic LDPred2-based MVP PGS only. We next performed other PGS evaluations with the selected OSA definition. The selected OSA definition was the one that had stronger association with the PGS.

#### Using TOPMed to identify an optimal multi-ethnic OSA PGS

Whole genome sequencing and quality control in TOPMed are described in Supplementary Note 4. We constructed the developed PGSs in TOPMed using PRCise 2 (v2.3.1. e; without any clumping nor thresholding). We estimated the association between the PGS and OSA in mixed models implemented in the GENESIS R package (version v2.16.1), with relatedness modelled via a sparse kinship matrix. All models were further adjusted for age, sex, BMI (linear and squared terms), self-reported race/ethnicity, and the first 11 principal components (PCs) of genetic data to prevent population stratification. We note here that we adjusted for self-reported race/ethnicity because health disparities related to the different sociocultural and structured environment experienced by different demographic groups may result in differences in OSA rates between them.^37^

We assessed OSA PGS in a multi-step process.

1. **Choosing a reference panel for ancestry-specific PGS development when using PRS-CS.** We compared PGS developed with our MVP LD reference panel to those developed using the existing UKBB LD reference panels. For this comparison we focused on ancestry-specific PGSs developed using the PRS-CS method. If UKBB reference panel performed better or equally well, we proceeded with the UKBB reference panel in PGS using the PRS-CS software, because the UKBB reference panel is publicly available and thus more useful to the research community.
2. **Compare ancestry-specific PRS-CS and LDPred2 PGSs.** We compared PRS-CS PGSs developed with the reference panel selected in step 1, with PGSs generated by LDPred2 using the MVP LD reference panel. For LDPred2 we only used the MVP reference panel because the software does not provide a reference panel.
3. **Compare PGS based on multi-ethnic meta-analysis to weighted ancestry-specific PGS combinations.** We compared PGSs constructed based on multi-ethnic meta-analyses and PGSs constructed as PGS combinations: combinations of PRS-CSx-derived PGSs, and combinations of either PRS-CS or LDPred2 PGSs according to step 2 comparison. We used both weighted and unweighted analyses.

As the conclusion of step 3, we selected the optimal multi-ethnic OSA PGSs based on BMI-adjusted and BMI-unadjusted analyses. When comparing PGSs, they were prioritised based on effect size estimates and p-values.

We differentiate the names of the two, final, selected OSA PGSs according to their determining factor: BMI adjustment, or lack thereof, of the OSA GWAS that were used as the foundation for the PGSs. Thus, we use the terms BMIadjOSA-PGS and BMIunadjOSA-PGS to address the selected, multi-ethnic, OSA PGSs that were developed based on GWAS that were, and were not, adjusted for BMI, respectively.

#### Secondary PGS comparisons and analyses in TOPMed

We performed additional comparisons with the multi-ethnic OSA PGSs: (a) we compared the selected multi-ethnic PGSs to ancestry-specific PGSs within relevant population groups, and (b) we studied the associations of sex-specific PGSs with OSA within sex groups and combined, to assess whether PGS developed based on sex-specific GWAS perform better within their corresponding sex groups, and whether the GWAS sample size is sufficient (especially for female participants) to develop sex-specific PGSs.

#### OSA and lung function PGS associations with OSA phenotypes and with other sleep phenotypes in TOPMed

We report the selected OSA PGS associations with OSA in TOPMed in the overall sample, stratified by self-reported race/ethnicity, sex, obesity status, and by age (≤40, 41–60, ≥61). In secondary analysis focussing on unrelated individuals due to model convergence issue, we further stratified to more granular BMI-defined levels of healthy BMI, overweight, and obese individuals.

Some of the TOPMed studies performed over-night sleep studies, and some administered sleep questions, enabling associations of selected OSA PGSs with additional sleep phenotypes. We estimated the association of the OSA PGSs with overnight sleep-evaluated phenotypes that rely on oxyhaemoglobin saturation and desaturation, and are typically associated with OSA. These phenotypes included AHI/REI, hypoxic burden,^38^ minimum and average oxyhaemoglobin saturation during sleep, average oxyhaemoglobin desaturation during respiratory events (i.e., desaturation compared to the baseline saturation at the beginning of the sleep period), and percent sleep time under 90% oxyhaemoglobin saturation. Some of these phenotypes were further evaluated separately during rapid and non-rapid eye movement (REM and NREM) sleep.^23^ For comparison, we also constructed a PGS for lung function and evaluated its association with some of these phenotypes. The lung function PGS was constructed based on PGS variants and weights downloaded from the PGS Catalogue,^39^ PGS ID PGS001180, based on the ratio between forced expiratory volume in 1s to forced vital capacity and developed by Tanigawa et al. (2022).^40^

We also estimated the association of the OSA PGSs with self-reported sleep phenotypes: sleep duration (harmonised in^41^), short and long sleep (defined as sleep duration <6 and >9 h, respectively), the Epworth sleepiness scale (ESS), including an excessive daytime sleepiness phenotype defined as ESS >10, and the Women Health Initiative Insomnia rating scale (WHIIRS), including an insomnia phenotype defined as WHIIRS ≥9. TOPMed studies contributing to each analysis are provided in Supplementary Table S2.

### OSA PGS association with OSA in Geisinger's MyCode

The MyCode Community Health Initiative (MyCode) Study is a hospital-based cohort study recruited from Geisinger, a large healthcare provider in central Pennsylvania. Subjects that have provided biospecimens have been genotyped through a collaboration with Regeneron Genetics Center as part of the DiscovEHR Study (http://www.discovehrshare.com/), including up to 170,765 participants available at the time of this analysis. All participants were genotyped using the Illumina's HumanOmniExpressExome (∼60,000) or Global Screening Array (∼110,000). All array-based data were quality controlled (QC'ed) with standard QC procedures before imputation, including gender mismatches, duplicate samples (identical twins were kept), low individual and SNP call rates (<95%), Hardy–Weinberg equilibrium (P ≤ 1 × 10^−15^), and heterozygosity (F > 0.4). QC'ed array data was imputed to the TOPMed Release 2 reference panel by array. Following imputation, all variants were filtered if they exhibited a minor allele count (MAC) of less than 5, a mean imputation quality score (R^2^) <0.7, or missingness >10%.

The current analysis was restricted to a subset of unrelated adult MyCode participants up to 2nd degree, selected using PRIMUS.^42^ OSA cases were defined based on a minimum of three OSA-related ICD-9 (ICD-9: 327.20, 327.23, 327.29, 780.51, 780.53, 780.57) or ICD-10 (G4730, G4733 or G4739) on separate dates, controls included those with zero instances of a relevant OSA ICD code, and those with one or two OSA codes were excluded from the analysis. This phenotype was based on a previous chart validation in the Geisinger study participants.^43^

BMIadjOSA- and BMIunadjOSA-PGSs were generated from files with SNP and weight using PRSice2 without clumping and thresholding. PGS associations with OSA were carried out both stratified and combined across sexes using GENESIS and AUC was estimated using pROC. All analyses were carried out using R. All association analyses were adjusted for age, BMI, BMI^2^, sex (in combined analysis), self-reported or EHR-derived race/ethnicity, and the top 20 genetic PCs.

### OSA PGS associations with outcomes in All of Us

We used WGS data from All of Us, version 6. Sequencing and quality control procedures for All of Us, performed by the All of Us team, are described in https://support.researchallofus.org/hc/en-us/categories/4537007565204-Genomics. The data was available in a HAIL matrix table. We used python version 3 on the All of Us Researcher Workbench. We first filtered genetic files to keep only HapMap SNPs, then converted the file to BED plink format. Next, we filtered out variants that failed All of Us quality control according to the “filter” flag, and variants with missing call rate >1%. We did not further filter by allele frequency. We constructed the PGSs selected by the TOPMed analysis using plink. We only used variants with MAF ≥0.01. In this dataset, we also compared the TOPMed-selected PGSs to previously-reported PGSs, in order to alleviate potential effects of overfitting, where TOPMed was used to select the main PGSs reported here. We estimated the associations of OSA PGSs with OSA and other clinical outcomes: hypertension, type 2 diabetes, stroke, atrial fibrillation, heart failure, coronary artery disease, asthma, chronic obstructive pulmonary disease, and chronic kidney disease. We performed sex-combined and sex-stratified analyses for all phenotypes, and for OSA, also analyses stratified by self-reported race/ethnicity, BMI categories (healthy BMI: BMI <25, overweight: BMI ≥25 and <30, and obese: BMI ≥30), age categories (age ≤40, 40 < age ≤ 60, age >60), smoking status (current, former, never smoker), alcohol drinking habits (never, former, moderate, binge and hazardous), and general self-reported rating of physical health (very good, fair, poor). We also performed a secondary analysis of the primary OSA PGS-OSA association adjusted for smoking status. Supplementary Note 5 provides the definitions of the phenotypes used (medical codes, etc.).

### OSA PGS associations across OSA and sleep measures in the Human Phenotype Project

We studied the association of OSA PGS with OSA, both from clinical diagnosis and from sleep monitoring, and with self-reported sleep measures in the HPP.^44^ Home sleep study was performed using the WatchPAT device (Itamar medical) over three nights. To determine OSA status based on sleep monitoring, we averaged the pAHI measures from all available nights and then used pAHI ≥15 as cutoffs for defining OSA, where pAHI is the peripheral arterial tonometry-derived AHI measured by WatchPAT. To assess potential association modification by relevant adjusting variables available in HPP, we performed a secondary analysis adjusting for smoking status and for waist circumference, an analysis further adjusting for neck circumference, and an analysis stratified by number of weekly days with moderate physical activity (0 days: not active, 1–3 days: moderately active, 4–7 days: active). We also estimated PGS associations with NREM and REM AHI, ODI, and RDI, and device-measured sleep duration, as continuous measures in association with the OSA PGS. Analyses were adjusted for age, sex, and BMI (linear and squared terms). Self-reported sleep phenotypes were available via questionnaires mimicking the UKBB project. We estimated the associations of OSA PGS with the responses to the following variables: “Daily sleep hours”, “Tired or little energy fortnight”, “Consider yourself morning evening”, “Easy getting up”, “Nap during day”, and “Trouble falling asleep”. We adjusted for the same variables as before.

### OSA PGS associations with body fat DXA measures in the Human Phenotype Project

We estimated the associations of the developed OSA PGSs with DXA measures available from the HPP. The relevant available measures Included VAT and SAT masses across the entire body (total scan), total mass, and gynoid and android masses, where gynoid correspond to the hips area and android to the waist area. We used 6 measures: ratios of VAT, SAT, gynoid, and android masses out of the total mass, and VAT:SAT and gynoid:android mass ratios. For each of the raw measures, we first checked for and removed any outliers. For distributions that appeared log-normal, we applied log transformation, and if the association results were the same (similar direction and p-value) as in the untransformed phenotype, we used the untransformed phenotype. We used only genetically unrelated individuals, performed BMi-adjusted and -unadjusted analysis using the same covariates as described in the previous section, and performed both sex-combined and sex-stratified analyses.

### Statistics

Analyses in MGB Biobank, used to develop multi-PRS combination weights, were logistic regressions adjusted for current age, sex, self-reported race/ethnicity, genotyping batch, and BMI (linear and squared terms), with BMI being the median BMI in the health records for each person. In TOPMed, we estimated the association between the PGS and OSA in mixed models (logistic when the outcome was binary and linear when the outcome was continuous) implemented in the GENESIS R package (version v2.16.1), with relatedness modelled via a sparse kinship matrix. All models were further adjusted for age, sex, BMI (linear and squared terms), self-reported race/ethnicity, and the first 11 principal components (PCs) of genetic data to prevent population stratification. In MyCode PGS associations with OSA were estimated using GENESIS and AUC was estimated using the pROC R function. All analyses were carried out using R. Association analyses were adjusted for age, BMI, BMI^2^, sex (in combined analysis), self-reported or EHR-derived race/ethnicity, and the top 20 genetic PCs. In All of Us, association analyses were carried out using R, and were adjusted for age (at the first code reporting the outcome in cases, current age for controls), BMI (linear and squared terms; BMI was taken as the nearest BMI from the EHR to the first code reporting the relevant outcome, and for controls BMI was taken as the most recent value), sex assigned at birth (for sex combined analysis), race/ethnicity (with categories being White, Black, Asian, Hispanic/Latino, and unknown/multi), and the first 10 genetic PCs. In HPP, analyses were adjusted for age, sex, and BMI (linear and squared terms), and the first 10 genetic PCs. In all cohorts/studies, secondary analyses did not adjust for BMI. Various stratified analyses and sensitivity analyses using additional adjusting covariates were performed in TOPMed, All of Us, and HPP, as described, including stratification of all analyses by sex. Throughout, all individuals with appropriate data were eligible to participate in the analysis. Individuals with missing data were excluded. In all studies, effect sizes and standard errors were obtained from the linear or logistic regression model (mixed regression in TOPMed), and we used the 1 degree of freedom Wald test for testing the null hypothesis of no association between the PRS and the relevant outcome.

We provide developed scripts used to perform analyses described in the paper and code to construct the OSA-PGSs in the GitHub repository https://github.com/nkurniansyah/OSA_PRS and the Zenodo repository https://doi.org/10.5281/zenodo.15547287.

### Ethics

MVP received ethical/study protocol approval from the VA Central Institutional Review Board, and written informed consent was obtained for all participants. Study subjects in FinnGen provided informed consent for biobank research, based on the Finnish Biobank Act. Full ethics statement is provided in Supplementary Note 6. The All of Us research program was approved by a single IRB, the “All of Us IRB”, which is charged with reviewing the protocol, informed consent, and other participant-facing materials for the *All of Us* Research Program. The IRB follows the regulations and guidance of the Office for Human Research Protections for all studies, ensuring that the rights and welfare of research participants are overseen and protected uniformly. More information is provided online https://allofus.nih.gov/about/who-we-are/institutional-review-board-irb-of-all-of-us-research-program. Ethics statement for MGB Biobank is provided in Supplementary Note 2, and for TOPMed studies in Supplementary Note 3. The Human Phenotype Project's data used in this work was approved by the Weizmann Institute's IRB for the 10K study, protocol 578-1. The MyCode Study was approved by the Geisinger Institutional Review Board and all participants provided informed consent. The current analysis consisted of secondary analysis of existing de-identified data and was deemed to be not “human subjects” research as defined in 45 CFR 46.102(f). This analysis was approved by the Beth Israel Deaconess Medical Center Committee on Clinical Investigations, protocol #2023P000279, and by the Mass General Brigham IRB, protocol #2021P001928.

### Role of the funder

As a study that used previously collected data, the funding source did not have any role in the study design, analysis, interpretation, or decision to submit this paper for publication.

## Results

We applied three modern Bayesian shrinkage methods (LDPred2, PRS-CS, and PRS-CSx) to develop PGSs based on GWAS summary statistics (Table 1) and meta-analysis combinations (ancestry-specific, multi-population, sex-specific), and further developed PGSs as combinations (sums) of other PGSs. Including, we performed a new GWAS of OSA in the FinnGen dataset, with a larger dataset compared to the published GWAS. Baseline characteristics of FinnGen participants are presented in Supplementary Table S3. We used the Mass General Brigham (MGB) Biobank dataset to train PGS summation weights (MGB participant characteristics are provided in Supplementary Table S4), the Trans-omics for Precision Medicine (TOPMed) dataset to assess PGS, and then data from All of Us (AoU), Geisinger, and HPP, to further study the selected PGSs in association with OSA and sleep-related measures, as well as other health outcomes.

### Evaluation of PGSs in TOPMed

The clinical characteristics of TOPMed participants are provided in Supplementary Table S5. Briefly, participants were self-identified from 4 race/ethnicity groups: White, Black, Hispanic/Latino, and Asian. Characteristics of individuals differed across these groups. For example, the average ages of participants were 68 years (Asian), 63 years (White), 56 years (Black), and 48 years (Hispanic/Latino). Most population groups had balanced sex ratio (50% female), while 58% of Hispanics/Latinos were female. Mean BMI was similar (∼30 kg/m^2^) in the group of White, Black, and Hispanic/Latino individuals, but lower (∼24 kg/m^2^) on average, in the group of Asian participants.

As described in Supplementary Note 1 and Supplementary Figures S1 and S2, using PRS-CS with the public UKB LD reference panel resulted in equivalent or better performance to PRS-CS using the MVP reference panel. Next, we compared LDPred2 (with MVP LD reference panel) to PRS-CS (with UKB reference panel) ancestry-specific PGSs. The results are provided in Supplementary Figures S3 and S4, demonstrating that the results were comparable for both approaches, except for White individuals; in this case, using the PRS-CS with UKB reference panel performed better. Therefore, for ancestry-specific PGSs we proceeded with PRS-CS with UKB LD reference panel. Next, we addressed the multi-ethnic PGS construction by comparing: (i) an LDPred2 PGS based on the multi-ethnic meta-analysis of all GWAS with the multi-population MVP reference panel, multi-PGS summation of either standardised PRS-CS or PRS-CSx ancestry-specific PGSs using either (ii) MGB-inferred weights, or (iii) equal weights. As shown in Supplementary Figures S5 and S6, the best performing PGSs were MGB-inferred weighted combination of PRS-CS PGSs. Here, the PGS based on BMI-adjusted GWAS, called BMIadjOSA-PGS, is the combination is of 5 PGSs, based on GWAS of: MVP White group, FinnGen, MVP Black group, MVP Hispanic/Latino group. The final PGS based on BMI-unadjusted GWAS, called BMIunadjOSA-PGS, is a weighted summation of 6 PGSs, based on the BMI-unadjusted versions of the above GWAS, and further including a PGS developed based on MVP Asian group meta-analysed with the Han Chinese OSA GWAS. The two final PGSs (BMIadjOSA-PGS and BMIunadjOSA-PGS) were highly correlated: Pearson correlation was 0.96, computed over the TOPMed dataset. Mean and standard deviations (SDs) of these PGSs computed over the TOPMed datasets are provided in Supplementary Table S6.

**Fig. 2:**
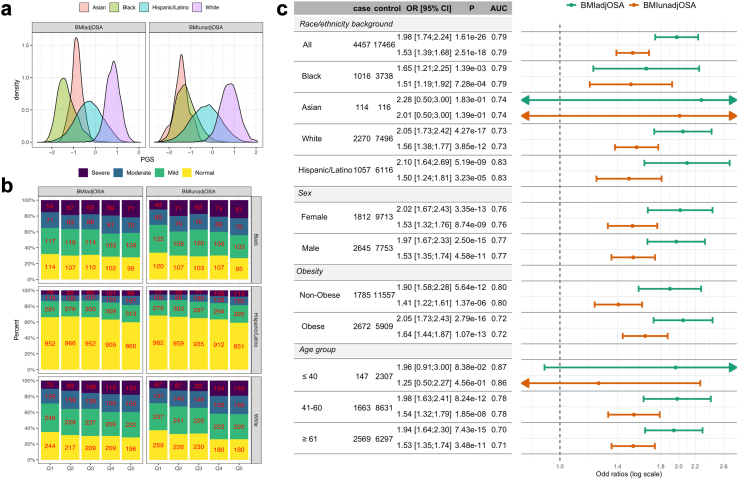
**OSA PGS associations with OSA in TOPMed individuals**. Panel a: distributions of BMIadjOSA- and BMIunadjOSA-PGSs in strata defined by self-reported race/ethnicity. Panel b: percentages and numbers of individuals with normal, mild, moderate, and severe OSA (defined by cut-points of REI/AHI of 5, 15, and 30), in quintiles of the OSA PGSs by self-reported race/ethnicity (Asian group excluded due to low sample size). Limited to individuals with measured REI/AHI. Panel c: estimated OSA PGS associations with OSA in TOPMed combined and stratified samples. Analyses were adjusted for age, sex (unless sex-stratified), self-reported race/ethnicity (unless stratified by that), and BMI (both linear and squared terms). AHI, apnoea-hypopnea index; OSA, obstructive sleep apnoea; PGS, polygenic score; REI, respiratory event index.

Fig. 2 compares the associations of the selected BMIadjOSA-PGS and BMIunadjOSA-PGS with OSA in TOPMed individuals, stratified by self-reported race/ethnicity, age, obesity status, and sex. Here, OSA was defined using the apnoea-hypopnea index (AHI) or the respiratory event index (REI), depending on availability of the measurement, or self-reported diagnosis of OSA (when overnight sleep study was not available, see Supplementary Table S4 for OSA definition by study). When using AHI or REI, OSA was defined as AHI/REI ≥15. The association models were adjusted for self-reported race/ethnicity, age, sex, BMI (both linear and squared terms), and the first 11 PCs of genetic ancestry. The OSA associations of the BMIadjOSA-PGS are stronger than the association of the BMIunadjOSA-PGS. In raw association, presented as proportions of individuals in OSA categories (no OSA, mild, moderate, and severe OSA) within quintiles of the PGS, both PGSs result in similar patterns (Fig. 2b). Due to the differences in PGS distribution between self-reported race/ethnicity groups (Fig. 2a), group-combined raw (quintile-based) associations are not appropriate; however, the PGS associations with OSA are very consistent across different race/ethnicity, sex, and age strata in covariate-adjusted models (Fig. 2c). Supplementary Figure S7 compares association results across more refine categories of unrelated healthy BMI, overweight, and obese individuals. While the association strength is not monotone across these strata, it is interesting to observe the strong associations between OSA PGSs and OSA in the healthy BMI stratum: BMIadjOSA-PGS OR is 2.39, 95% CI: [1.67; 3.42].

**Fig. 3:**
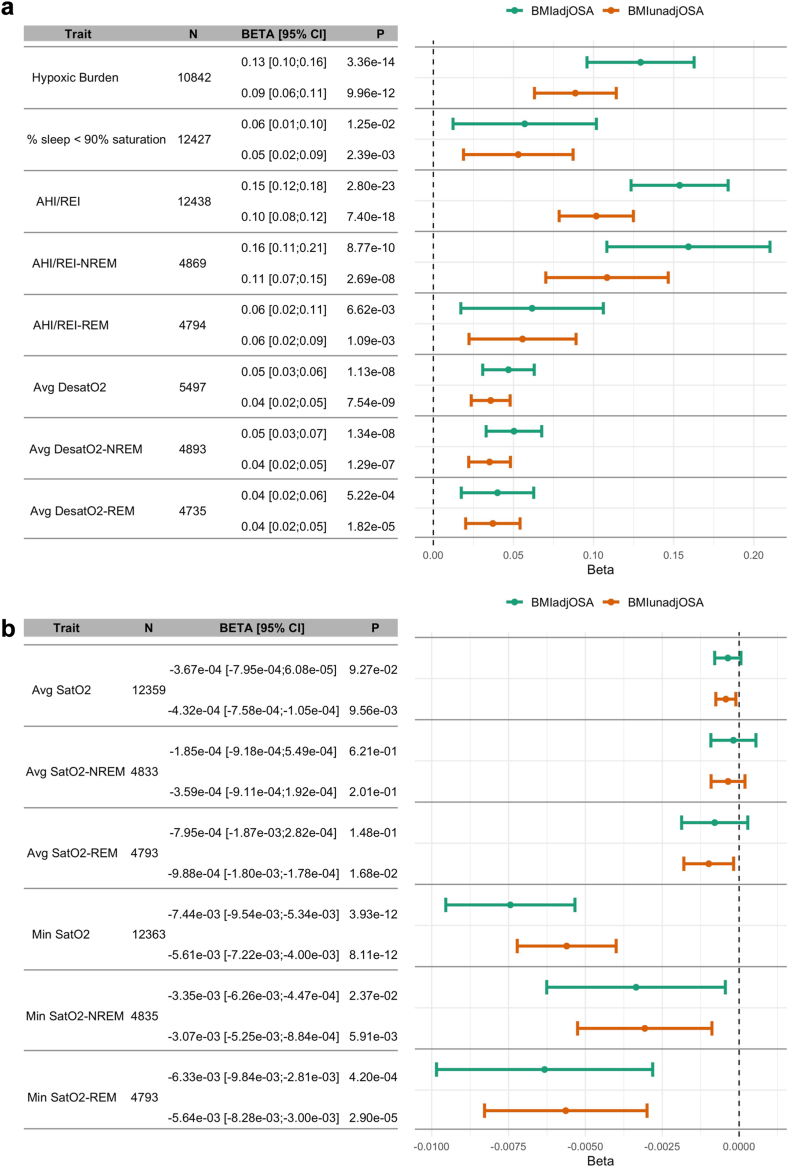
**Estimated associations of OSA PGSs with OSA- and sleep-related phenotypes in TOPMed**. Results from association analyses of OSA PGSs with OSA-related sleep measures. Panel a: measures that tend to be higher with more severe OSA, panel b: measures that tend to be lower with more severe OSA. AHI, apnoea-hypopnea index; NREM, non-rapid eye movement sleep; REI, respiratory event index; REM, rapid eye movement sleep; Avg SatO2, average oxyhaemoglobin saturation during sleep; Min SatO2, minimum oxyhaemoglobin saturation during sleep; Avg DesatO2, average oxyhaemoglobin desaturation during respiratory events.

In TOPMed individuals who had OSA and OSA-related phenotypes available from overnight sleep testing, analyses using both BMIadjOSA- and BMIunadjOSA-PGSs (Fig. 3) were generated using participants whose phenotypic characteristics are reported in Supplementary Tables S7 and S8 The PGSs are generally associated with OSA phenotypes (AHI, hypoxic burden, percent sleep time with oxyhaemoglobin saturation below 90%, minimum and average oxygen saturation, and respiratory event-related oxygen desaturation during sleep; all log transformed), and are in the expected direction of effect. There are differences, however, in the trait associations between REM and NREM sleep, with stronger associations in phenotypes measured during NREM sleep (e.g. AHI: 0.15, 95% CI: [0.12, 0.18]; NREM AHI: 0.16, 95% CI: [0.11, 0.21]; REM AHI: 0.06, 95% CI: [0.02, 0.11]). In addition, the PGS association with average oxyhaemoglobin saturation during sleep (Avg SatO2) is very weak (effect size: −0.0003, 95% CI overlapping with zero, p-value = 0.09). To address the hypothesis that this result is because Avg SatO2 potentially reflects lung function rather than OSA, we estimated the association of lung function (FEV1 to FVC ratio) PGS with Avg SatO2 as well as with AHI, both log transformed. The lung function PGS was associated with Avg SatO2 (effect size: −0.0002 percent, 95% CI: [−0.003, −0.00007], p-value = 9.0 × 10^−4^) but only weakly with AHI (effect size: 0.01 events per hour, 95% CI: [0.002, 0.2], p-value = 0.02, Supplementary Table S9).

Sex specific PGSs (based on sex-specific GWAS) did not exhibit better performance than PGSs based on sex-combined analysis (Supplementary Figures S8 and S9).

### Associations of OSA PGSs in the Geisinger's MyCode project

**Fig. 4:**
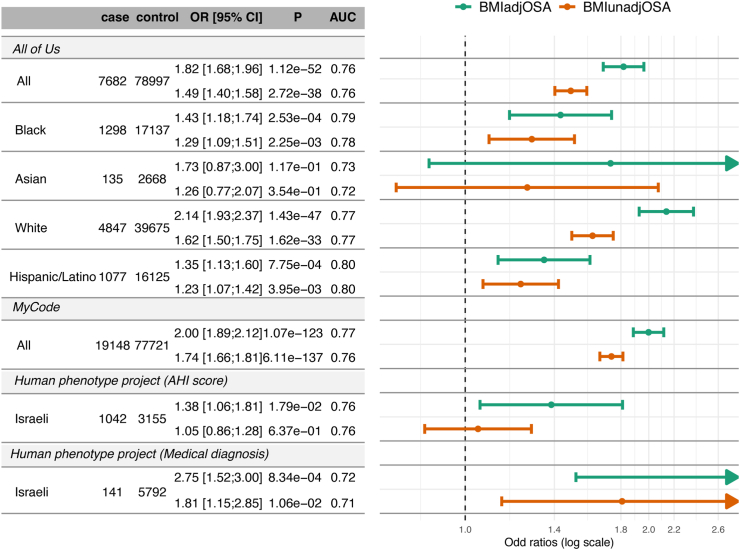
**OSA PGS associations with OSA in validation studies**. Estimated associations of BMIadjOSA- and BMIunadjOSA-PGSs with OSA in three validation datasets: All of Us, Geinsinger health system's MyCode, and the Human Phenotype Project, in combined and stratified analyses. BMI, body mass index; OSA, obstructive sleep apnoea; PGS, polygenic score.

The MyCode project include individuals recruited from the Geinsinger healthcare system. OSA categorization is based on ICD codes, as described in the Methods section. Fig. 4 provides results from association analyses of BMIadjOSA-PGS and BMIunadjOSA-PGS in MyCode in a sex-combined analysis. Results from sex-stratified analysis are provided in Supplementary Figure S10. Characteristics of the study population are provided in Supplementary Table S10. There were 96,869 individuals, of which 19,148 (19.8%) had OSA. The mean age was 48 years, and 60% were female. Majority of individuals were White. The OSA PGSs were highly associated with OSA: BMIadjOSA-PGS overall OR = 2.00, 95% CI: [1.89; 2.12], in females OR = 1.89, 95% CI: [1.75; 2.05], and in males OR = 2.14, 95% CI: [1.97; 2.23].

### Associations of OSA PGSs with OSA phenotypes in the Human Phenotype Project

Supplementary Table S11 characterises the participants from the HPP. There were 3070 female and 2880 male participants, ∼52 years old on average, and the average BMI was ∼26 kg/m^2^. Very few individuals had OSA according to medical diagnosis: 0.75% of female and 4.1% of male participants. Supplementary Table S12 further characterises the subset of individuals who had sleep monitoring data measured using the WatchPAT device, and their objective sleep phenotypes (averaged values over three nights). Based on sleep monitoring data, 17.2% of female and 30.7% of male individuals had OSA defined as pAHI ≥15. BMIadjOSA-PGS was strongly associated with OSA in the HPP (Fig. 4). The association was stronger when OSA was based on medical diagnosis (141 cases, 5792 controls; OR = 2.75), compared to the association when OSA was defined based on a 3-night average of pAHI ≥15 (1042 cases, 3155 controls; OR = 1.38). As reported in Supplementary Figure S11, associations slightly weakened in association models that further adjusted for waist circumference and smoking status (BMIadjOSA-PGS OR = 1.34, 95% CI: [0.99; 1.81] for OSA based on pAHI, BMIadjOSA-PGS OR = 2.27, 95% CI: [1.26; 4.11] for OSA based on clinical diagnosis from health records). In contrast, associations effect sizes increased in a model further adjusting for neck circumference (BMIadjOSA-PGS OR = 2.57, 95% CI: [1.17; 5.66] for OSA based on pAHI, BMIadjOSA-PGS OR = 3.68, 95% CI: [0.74; 1767] for OSA based on clinical diagnosis from health records). However, we note that the sample sizes reduced substantially when adjusting to neck circumference. Supplementary Figure S12 presents analysis stratified by levels of physical activity, there was a monotone increasing relationship between the estimated effect sizes of the OSA PGSs with increasing levels of physical activity. Focussing on BMIadjOSA-PGS, OR was 1.23, 95% CI: [0.69; 2.18] in inactive individuals, and 2.19, 95% CI: [1.16, 4.12], in active individuals.

**Fig. 5:**
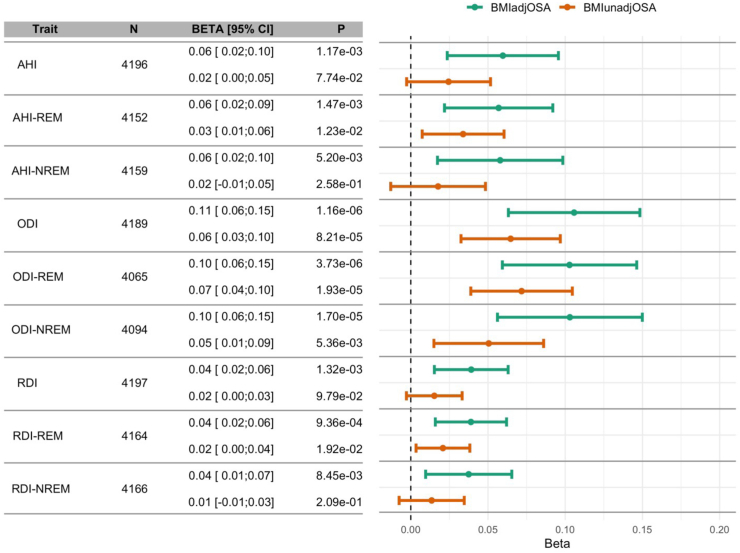
**OSA PGS associations with quantitative OSA phenotypes in the Human Phenotype Project**. Estimated associations of BMIadjOSA-PGS and BMIunadjOSA-PGS with sleep monitoring, log-transformed OSA-related measures in the HPP, stratified by REM and NREM sleep and combined. All measures were averaged over the three nights of sleep monitoring. AHI, peripheral arterial tonometry-derived apnoea-hypopnea index; HPP, Human Phenotype Project; ODI, oxygen desaturation index; RDI, respiratory disturbance index; REM, rapid eye movement; NREM, non-REM.

Unlike the analysis in the TOPMed data, associations of quantitative OSA-related phenotypes with the OSA PGSs were similar when stratified by REM and NREM stage (Fig. 5). PGS associations were stronger with the oxygen desaturation index (ODI) compared to the pAHI or respiratory disturbance index (RDI). We also estimated associations with oxygen saturation phenotypes (Supplementary Figure S13), sleep stage duration (percentages of total sleep duration) (Supplementary Figure S14), and with self-reported sleep phenotypes (Supplementary Figure S15). OSA PGSs were strongly associated with lower minimum and average oxygen saturation during sleep, with stronger associations for BMIadjOSA-PGS compared to BMIunadjOSA-PGS. The PGS associations with other phenotypes were generally weaker. Among the associations that had p-value <0.05, BMIunadjOSA-PGS was associated with less sleep time percentage in light sleep and longer percentage of time in REM sleep. BMIadjOSA-PGS was weakly associate with higher level of daytime sleepiness (beta from association with a continuous, Likert scale response = 0.05, p-value = 0.04) and BMIunadjOSA-PGS was associated with both shorter sleep duration (beta from association with continuous sleep hours question = −0.07, p-value = 0.03), and with lower likelihood of long (>9 h) sleep duration (OR = 0.35 ≤9 h, p-value = 0.04). Characteristics of self-reported sleep phenotypes among the HPP participants are provided in Supplementary Table S13.

### Associations of OSA PGSs with clinical outcomes in the All of Us study

All of Us participant characteristics are provided in Supplementary Table S14. Between 6% and 15% of participants were classified with OSA, with the lowest and highest percentages observed in self-reported Asian and White participants, respectively. All association analyses were adjusted for age, BMI (linear and squared terms), sex at birth, self-reported race/ethnic background, and the first 10 genetic PCs. First, we performed OSA association analysis in the combined cohort with BMIadjOSA-PGS, BMIunadjOSA-PGS, as well as with other previously reported PGSs that used either only genome-wide significant SNPs,^21^ or used the clump & threshold method to derive PGS based on BMI-unadjusted analysis using FinnGen-only data.^10^ The results are provided in Supplementary Table S15, demonstrating that the newly-developed OSA PGSs have stronger associations with OSA compared to previously-developed ones. Fig. 4 visualises the associations of the developed OSA PGSs with OSA in All of Us (as well as other cohorts), and Supplementary Figure S7 provides results stratified by levels of BMI (together with TOPMed results, for comparison), Supplementary Figure S10 provides results stratified by sex and age (together with MyCode results, for comparison), and Supplementary Figure S16 provides results stratified by smoking status, alcohol drinking habits, and general self-reported physical health. Unlike in TOPMed, the OSA PGS association estimates in All of Us differed across self-reported race/ethnicity groups, with the association in self-reported White individuals being the strongest (OR = 2.14 of BMIadjOSA-PGS, 95% CI: [1.93, 2.37]), and weakest in self-reported Hispanic/Latino individuals (BMIadjOSA-PGS OR = 1.35, 95% CI: [1.13, 1.60]). There were some differences in association effect sizes across other strata as well, with often the strongest association observed in the healthiest group. For example, BMIadjOSA-PGS OR was 2.13, 95% CI: [1.68; 2.70]. In the normal weight groups, in contrast to 1.72, 95% CI: [1.57; 1.90] in the obesity groups. Similarly, BMIadjOSA-PGS OR was 2.17, 95% CI: [1.93; 2.43] in the group with very good self-reported physical health, in contrast to OR of 1.65, 95% CI: [1.35; 2.02] in the group with poor self-reported physical health. Patterns were less clear when considering smoking and alcohol stratifications.

**Fig. 6:**
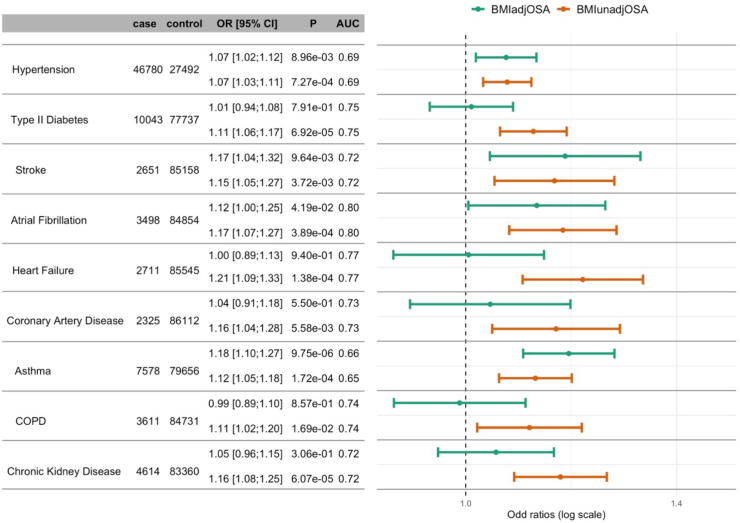
**OSA PGS associations with other clinical outcomes in All of Us**. Estimated associations of BMIadjOSA and BMIunadjOSA PGSs with clinical outcomes in the All of Us study. Associations were adjusted for age, sex, BMI (linear and squared terms), and 10 PCs of genetic ancestry. BMI, body mass index; COPD, chronic obstructive pulmonary disease; OSA, obstructive sleep apnoea; PGS, polygenic score; PC, principal component.

Next, we performed association analyses of BMIadjOSA-PGS and BMIunadjOSA-PGS with clinical outcomes potentially related to OSA: hypertension, type 2 diabetes, stroke, atrial fibrillation, heart failure, coronary artery disease, asthma, chronic obstructive pulmonary disease (COPD), and chronic kidney disease. The results are visualised in Fig. 6. BMIunadjOSA-PGS was associated with all clinical cardiovascular, pulmonary, and kidney outcomes, while BMIadjOSA-PGS had more variable, and usually weaker, associations. Notably, BMIadjOSA-PGS was not associated with type 2 diabetes, heart failure, coronary artery disease, COPD, or chronic kidney disease, but was associated with hypertension, stroke, atrial fibrillation, and asthma (raw p-value <0.05).

**Fig. 7:**
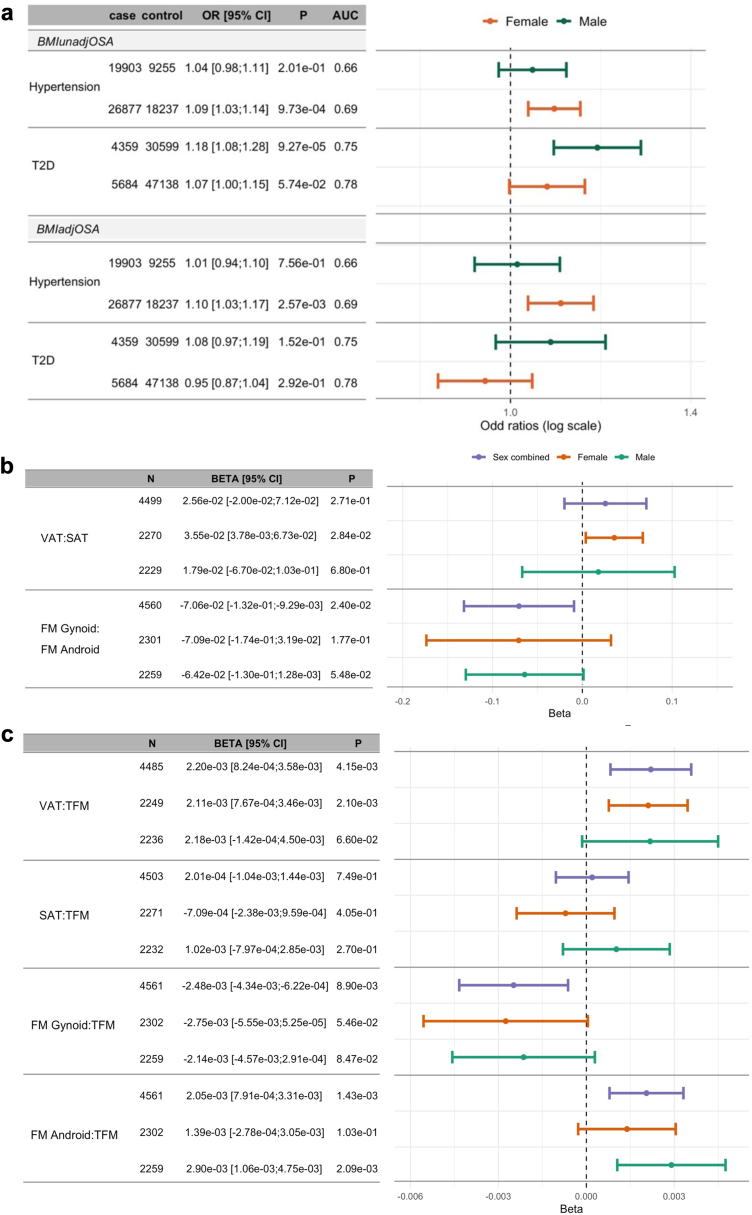
**Sex-stratified associations OSA PGSs with cardiometabolic traits and body fat distribution measures from DXA scan**. Panel a: Estimated adjusted odds ratio of OSA PGSs with hypertension and T2D in All of Us. Panels b and c: estimated associations of BMIadjOSA-PGS with DXA measures in HPP. All associations were adjusted for age, sex, BMI (linear and squared terms), and the 10 first genetic principal components. Results from association analyses that used BMIunadjOSA-PGS and was not adjusted for BMI are provided in Supplementary Figure S18. BMI, body mass index; DXA, dual-energy X-ray absorptiometry; HPP, human phenotype project; OSA, obstructive sleep apnoea; PGS, polygenic score; OR, odds ratio; VAT, visceral adipose tissue; SAT, subcutaneous adipose tissue; FM, fat mass; TFM, total scan fat mass; T2D, type 2 diabetes.

Fig. 7 (hypertension and type 2 diabetes) and Supplementary Figure S17 (all other outcomes) provide results from sex-stratified association analyses of OSA PGSs. The associations with hypertension and diabetes suggest sex-differences: Both BMIadjOSA-PGS and BMIunadjOSA-PGS associations with hypertension were driven by the female stratum, while the BMIunadjOSA-PGS association with T2D was driven by the male stratum.

### Associations of OSA PGSs with body fat distribution measures from DXA scan

We used data from the HPP to study whether BMIadjOSA and BMIunadjOSA PGSs are associated with body fat distribution measures to potentially explain their different associations with health outcomes. We considered measures based on total body visceral adipose tissue (VAT), subcutaneous adipose tissue (SAT), gynoid fat mass, and android fat masses. Specifically, we used, as outcomes, the proportions of total body VAT and SAT mass out of total body fat mass (TFM), the ratio VAT:SAT mass, the proportions of gynoid and android fat out of TFM, and the ratio gynoid:android fat mass. Characteristics of these DXA phenotypes are provided in Supplementary Table S16. Association analyses results are provided in Fig. 7 (BMI adjusted analyses) and Supplementary Figure S18 (BMI unadjusted analyses). In combined sex association analyses that were not adjusted for BMI, BMIunadjOSA-PGS was associated with all DXA outcomes. Higher BMIunadjOSA-PGS values were strongly associated with higher proportions of VAT and SAT mass proportions, and with higher android mass proportion but with lower gynoid mass proportion. Also, it was associated with increased VAT:SAT ratio, and with lower gynoid:android mass ratio. In BMI-adjusted analyses, results were similar, however, BMIadjOSA-PGS was not associated with SAT mass proportion. Further, BMIadjOSA-PGS was associated with VAT:SAT only in the female stratum (Fig. 7).

## Discussion

This is the largest study to date leveraging polygenic scores across global populations to evaluate both BMI-dependent and BMI-independent genetic risk for OSA and related outcomes. Associations of the BMIadjOSA-PGS with OSA, in BMI-adjusted analyses, were strong, and ranged from ORs of 1.38 for device-measured AHI ≥15 and 2.75 for medical diagnosis-based OSA in HPP, to 1.98 in All of Us, and 2.00 in MyCode, likely reflecting a more severe phenotype that triggered clinical recognition in the latter cohorts, as compared to OSA identified by general population screening in the former. Importantly, associations differed between PGS developed based on BMI-adjusted and BMI-unadjusted GWAS. Using data available from the All of Us cohort, we further showed that the strength of associations with cardiovascular, metabolic, pulmonary and kidney disorders often differed depending on the BMI adjustment of the source OSA GWAS. For example, type 2 diabetes, coronary artery disease, and heart failure associations were null in association with BMIadjOSA-PGS but were positively associated with BMIunadjOSA-PGS, suggesting that much of the co-aggregation of those cardio-metabolic phenotypes may be explained by genetic risk factors associated with obesity. In contrast, both BMIadjOSA- and BMIunadjOSA-PGSs were associated with hypertension, stroke, atrial fibrillation, and asthma, suggesting that obesity-independent risk factors for OSA are associated with those outcomes. Interestingly, epidemiological studies that adjusted for BMI and other cardiovascular confounders have generally shown more consistent associations with OSA for hypertension and stroke in comparison to coronary artery disease.^45^^,^^46^ These results confirm past findings of BMI-independent genetic basis of OSA from family studies,^47^ and point to a unique pathophysiology of OSA that may not be associated with elevated BMI, such as BMI-independent effects of OSA on autonomic regulation.

The association between asthma and BMIadjOSA-PGS is of interest given the reported associations between OSA and asthma phenotypes but uncertainty regarding the causal directionality of the relationship between OSA and asthma.^48^^,^^49^ Common risk factors for both disorders include not only obesity but also inflammation and atopy. Our study findings suggest that additional OSA-related risk factors, beyond obesity, may influence risk of asthma.

Obesity is one of the strongest risk factors for OSA and is independently an important risk factor for many other health outcomes including diabetes and various cardiovascular outcomes. Studies assessing the effect of OSA, and the effect of OSA treatment, on cardiovascular outcomes typically attempt to account for the role of BMI or obesity and evaluate the OSA-specific effect.^50^, ^51^, ^52^ Hence it is important to account for BMI in the development of OSA PGSs. The developed PGSs, BMIadjOSA-PGS and BMIunadjOSA-PGS, are highly correlated, yet have different associations with OSA and with other health outcomes. The BMIadjOSA-PGS has stronger association with OSA in BMI-adjusted analysis, while the BMIunadjOSA-PGS has stronger association with other outcomes, likely due to it capturing BMI-related OSA genetic effects. Both PGSs are useful and it would be incorrect to dismiss the BMIunadjOSA-PGS as capturing primarily obesity rather than OSA genetics, because the impact of obesity may be mediated through OSA. BMIadjOSA-PGS captures OSA genetic effects without the contribution of obesity. While it is difficult to assess how effective this adjustment is (i.e., does some of the obesity-related OSA genetics may remain captured in BMIadjOSA-PGS), the observed null association of BMIadjOSA-PGS with diabetes in All of Us suggests that it was largely effective. We studied the associations of OSA-related phenotypes with BMIadjOSA- and BMIunadjOSA-PGSs in both TOPMed and HPP, both while adjusting the associations to BMI. In TOPMed, the associations of oxygen saturation phenotypes were stronger with BMIunadjOSA-PGS compared to BMIadjOSA-PGS (but this was not replicated in HPP). More work is needed to assess whether this points to OSA-specific obesity-related pathophysiology, such as obesity–hypoxaemia interactions,^49^ or obesity-related reduction in lung volumes, which will contribute to both hypoxaemia and OSA severity.^53^^,^^54^ In a parallel investigation, secondary stratified analyses by BMI levels in TOPMed and All of Us suggest that OSA PGSs have stronger associations in individuals with health BMI (<25). It is possible that genetic determinants of OSA are more important (i.e., more expressed) in the absence of obesity and overweight.

We performed association analyses of OSA PGSs with DXA scan measures in the HPP. While it would have been ideal to consider fat measures separately in the neck/upper airway area^55^^,^^56^ and in the abdomen,^57^ we had overall VAT and SAT mass measures, and gynoid and android fat masses (not limited to VAT or SAT tissues). OSA PGSs were associated with higher VAT:SAT ratio, but the associations were statistically significant only in females (Fig. 7). This is consistent with sex differences in cardiometabolic risk conferred by VAT^58^ identified in the Framingham Heart Study. Higher VAT is associated with increased inflammation and insulin resistance,^59^^,^^60^ and increased insulin levels are in turn associated with OSA—even after adjusting for BMI.^61^ Insulin levels may be linked to OSA via the association of VAT with upper airway size and function.^62^ The association of OSA PGSs with VAT:SAT ratio, even in BMI-adjusted analysis, is consistent with the role of inflammation and insulin resistance in OSA. The stronger association observed in women also suggests a potential sex-specific mechanism.

Sex differences in OSA are well recognised, with differences in both pathophysiology (e.g. respiratory event duration) and symptoms of OSA (daytime sleepiness, co-morbid insomnia, snoring).^63^ Our results suggest that a sex-combined PGS, rather than sex-specific PGSs, is appropriate for OSA (under the limitation that data are more limited in sex strata, potentially reducing power of sex-specific PGSs). Yet, we saw sex differences in sex-specific associations of the OSA PGSs with outcomes particularly for metabolic outcomes that may be related to adiposity. While OSA PGS associations with OSA were very similar across sex strata in TOPMed (OSA mostly assessed using home sleep apnoea tests), PGS associations with OSA were weaker in females compared to males in both AoU and MyCode. In AoU, there were also some differences in OSA PGS associations with metabolic outcomes—T2D and hypertension. The association of OSA PGSs with hypertension was stronger in females, and, when assessing BMIadjOSA PGS, the association with hypertension was essentially null in males (OR = 1.01) while statistically significant in women (OR = 1.1, 95% CI: [1.03, 1.17]). While sex-specific associations of BMIadjOSA-PGS was T2D were not statistically significant, the association of BMIunadjOSA-PGS with males was stronger than that in females (male OR: 1.18, 95% CI: [1.08, 1.28]; female OR: 1.07, 95% CI: [1.00, 1.15]), despite stronger statistical power in females due to higher sample size and more T2D cases. In HPP, the association of BMIadjOSA-PGS with VAT:SAT is statistically significant only in females. These observed sex differences may point to differences in sex differences in genetic determinants of fat depots distribution in males and females, potentially explaining some of the known sex differences in metabolic syndrome and dysfunctional adiposity.^64^

Strengths of this study include the use of multiple independent and population-diverse datasets, with populations reflecting the diversity of the U.S. population, as well as a few world-wide populations. Our study is unique in that we employed both BMI-adjusted and -unadjusted GWAS to develop and assess PGSs, assessed OSA associations in a stratified manner, and estimated the associations of OSA PGSs with multiple clinical measures across available datasets. Our results suggest areas for improvement in future studies. Importantly, there remains the challenge posed by the underdiagnosis of OSA,^65^ limiting results from GWAS as well as estimations of associations.^66^ For example, the OSA PGSs effect size estimates demonstrated unprecedented consistency across strata defined by age, sex, and self-reported race/ethnicity (unlike previous results with blood pressure PGS^15^) in TOPMed, where most of the cohorts assessed OSA using objective devices. In contrast, associations differed by self-reported race/ethnicity in All of Us, where OSA status was determined by electronic health record codes. There, the associations were strongest in self-reported White populations. Well-known health disparities between White and minority populations in the U.S.,^67^ suggest the possibility of referral bias resulting in increased diagnostic misclassification of OSA in race/ethnic minority populations in the U.S. Such differences may underlie the observed differences in OSA PGS effect estimates, given the similarity of PGS effect sizes in cohorts utilizing a uniform screening procedure for OSA in TOPMed. This points to the potential utility of PGS to identify individuals at increased OSA risk in health disparity population groups. However, it has been consistently shown that PGS performance tends to be higher in populations that are genetically similar to their training populations.^68^ While we used as diverse a sample as possible, it is still dominated by individuals of European genetic ancestry. Thus, genetic studies of OSA, and consequently, PGSs of OSA, are still limited by both underdiagnosis of OSA and the lack of phenotyping across diverse populations, and these limitations disproportionally affect some population subgroups.

In summary, we developed BMIadjOSA- and BMIunadjOSA-PGSs, which were highly associated with OSA across multiple, diverse, populations. Follow up association analyses in All of Us revealed a genetically determined, OSA-specific, obesity-independent, association with hypertension, stroke, and asthma. The results support the importance of both obesity related and unrelated genetic risk factors for OSA. Future work should assess the potential use of OSA PGSs for OSA screening, especially in individuals who are very healthy (low BMI and having good physical health status) and combine genetic analysis methods with detailed phenotyping and endotyping data to create PGSs that correspond to OSA subphenotypes.

## Contributors

NK and TS conceptualised the study. NK performed data analysis in MVP, TOPMed, MGB biobank, and All of Us. YH performed data analysis in the HPP. SJS performed OSA GWAS in FinnGen. SG contributed to phenotype extraction and generation in MyCode. AEJ and GC performed PGS association analyses in MyCode. NK finalised all tables and figures. NK, BEC, BS, HW harmonised phenotypes in TOPMed. MM, BH, SYJ, RK, JIR, SSR, SAG, TMB, MF, AM, HMO-B, BMP, MF, LH, and SR contributed to study design, data collection, and data management, in TOPMed cohorts they represent. JH, PW, KC, and DJG contributed to study design and data management in MVP. AEJ, BTK and AP contributed to Geisinger study design and data management. HMO contributed to FinnGen study design and data management. NK, SJS, AEJ, and TS draughted the manuscript. TS supervised the work. NK, SJT, GC, SG, AEJ, YH, BTK, BEC, BWS, HW, JH, MRM, BH, SYJ, LMR, RK, JIR, SSR, SAG, TMB, PYL, HC, MF, LH, DL, AM, HMO-B, BMP, PWFW, AIP, HMO, SR, DJG, and TS all critically reviewed the manuscript and approved its final version. NK and TS directly accessed and verified the underlying data. FinnGen consortium member were involved in the FinnGen study design, data collection and curation. TOPMed consortium members were involved in the TOPMed study design, data collection and curation across the participating studies. The VA MVP program collected, organized, and curated data, and established computational platform to study health outcomes and their determinants in MVP participants.

## Data sharing statement

Summary statistics from BMI-adjusted and BMI-unadjusted OSA GWAS, stratified by sex and by HARE group, are available on dbGaP, study accession phs001672.

Individual-level genotypes and register data from FinnGen participants can be accessed by approved researchers via the Fingenious portal (https://site.fingenious.fi/en/) hosted by the Finnish Biobank Cooperative FinBB (https://finbb.fi/en/).

Full summary statistics for the genome-wide association study can be accessed from https://figshare.com/ (https://doi.org/10.6084/m9.figshare.20033246) and Bio-X institutes website (http://analysis.bio-x.cn/gwas/).

MGB Biobank genotyping and phenotypic data are available to Mass General Brigham investigators with required approval from the Mass General Brigham Institutional Review board (IRB).

BMIadjOSA-PGS and BMIunadjOSA-PGS variant and weights will be deposited to the PGS Catalogue. There are also available on the GitHub repository https://github.com/nkurniansyah/OSA_PRS. We also used additional PGSs for performance comparisons. All are available on the PGS catalogue (LDPred2 OSA PGS from Zhang et al., 2022: PGS003479, genome-wide significant variants OSA PGS based on BMI-adjusted MVP GWAS: PGS003858, genome-wide significant variants OSA PGS based on BMI-unadjusted MVP GWAS: PGS003857, pulmonary function PGS: PGP000244).

TOPMed freeze 8 WGS data are available by application to dbGaP according to the study specific accessions: ARIC: “phs001211“, CFS: “phs000954”, CHS: “phs001368”, COPD- Gene: “phs000951”, FHS: “phs000974”, HCHS/SOL: “phs001395”, JHS: “phs000964”, MESA: “phs001416”, WHI: “phs001237”. Phenotype data are available from dbGaP according to the study-specific accessions: ARIC: “phs000280“, CFS: “phs000284”, CHS: “phs000287”, COPD- Gene: “phs000179”, FHS: “phs000007”, HCHS/SOL: “phs000810”, JHS: “phs000286”, MESA: “phs000209”, WHI: “phs000200”.

Data from the NIH All of Us study are available via institutional data access for researchers who meet the criteria for access to confidential data. To register as a researcher with All of Us, researchers may use the following URL and complete the laid out steps: https://www.researchallofus.org/register/. Researchers can contact All of Us Researcher Workbench Support at support@researchallofus.org.

Data in this paper is part of the Human Phenotype Project (HPP) and is accessible to researchers from universities and other research institutions at: https://humanphenotypeproject.org/data-access. Interested bona fide researchers should contact info@pheno.ai to obtain instructions for accessing the data.

MyCode data can be accessed by Geisinger investigators. There are restrictions to the sharing of MyCode DiscovEHR genetic datasets related to agreements between Geisinger and the Regeneron Genetics Center.

## Declaration of interests

Dr. Cade reports receiving grants from the National Institute of Health (NIH) and from the American Academy of Sleep Medicine Foundation, and an unpaid consultancy, with a paid consulting agreement in progress through the institution, to Apnimed. Dr. Chen reports receiving consulting fees from Character Biosciences. Dr. Gottlieb reports receiving personal consulting fees from Powell Mansfield, Inc., Lilly USA, LLC, and Takeda Development Center Americas, Inc. He also reports receiving lecture honoraria from SleepRes, Inc, and from ProSomnus Sleep Technologies, and participation on a Data Safety Monitoring Board or Advisory Board for SleepRes, Inc and ProSomnus Sleep Technologies. Dr. Gupta held investment stocks of Eli Lilly (purchased in February 2024 and sold in January 2025) and of Regeneron (purchased in October 2024). Dr. Haring reports receiving lecture fees from Bristol Myers Squibb, Inari. Boehringer Ingelheim, and Pfizer, unrelated to the content of this manuscript. Dr. Keenan reports receiving support from grant P01HL160471 (Developing a P4 Medicine Approach to Obstructive Sleep Apnoea). Dr. Levy reports receiving honoraria for journal editing, as the editor in chief for IJC Cardiovascular Risk and Prevention. Dr. Moll reports receiving NIH grant K08HL159318, and a Genentech sponsored research agreement, with payments made to the institution. He also reports receiving consulting fees from 2ndMD, TheaHealth, Axon Advisors, Dialectica, Sanofi, and Verona Pharma, with payments made to him. Dr. Moll further reports payments or honoraria and travel support for lectures at the ATS 2024 and NYSTS 2024 conferences, with payments made to him. Dr. Psaty reports receiving NIH grant support, as reported in the CHS study acknowledgements, participation in the Steering Committee of the Yale Open Data Access Project, funded by Johnson & Johnson, and serving as a chair of the Board of Directors of the Am J Hypertension. Dr. Raffield reports receiving consulting fees as a consultant to the TOPMed Administrative Coordinating Center via Westat®. Dr. Redline reports receiving consulting fees from Eli Lilly, related to work on GLP-1 and OSA, with payments made to her. Dr. Rich reports receiving consulting fees from Westat, as a consultant to the Administrative Coordinating Center for the NHLBI Trans-Omics for Precision Medicine program. Dr. Rotter reports NIH grant support. Dr. Sofer reports grant support from the National Institute on Aging and from the National Heart Lung and Blood Institute, with payments made to the institution.
