# Supplementary material for: Polygenic scores for obstructive sleep apnoea reveal pathways contributing to cardiovascular disease
Source: eBioMedicine. 2025 Jun 4;117:105790. doi: 10.1016/j.ebiom.2025.105790 (PMC12171572; doi:10.1016/j.ebiom.2025.105790)
Supplement: Supplementary File 2 [file mmc2.docx]

| **First names** | **Surnames** |
| --- | --- |
| Namiko | Abe |
| Gonçalo | Abecasis |
| Francois | Aguet |
| Christine | Albert |
| Laura | Almasy |
| Alvaro | Alonso |
| Seth | Ament |
| Peter | Anderson |
| Pramod | Anugu |
| Deborah | Applebaum-Bowden |
| Kristin | Ardlie |
| Dan | Arking |
| Donna K | Arnett |
| Allison | Ashley-Koch |
| Stella | Aslibekyan |
| Tim | Assimes |
| Paul | Auer |
| Dimitrios | Avramopoulos |
| Najib | Ayas |
| Adithya | Balasubramanian |
| John | Barnard |
| Kathleen | Barnes |
| R. Graham | Barr |
| Emily | Barron-Casella |
| Lucas | Barwick |
| Terri | Beaty |
| Gerald | Beck |
| Diane | Becker |
| Lewis | Becker |
| Rebecca | Beer |
| Amber | Beitelshees |
| Emelia | Benjamin |
| Takis | Benos |
| Marcos | Bezerra |
| Larry | Bielak |
| Joshua | Bis |
| Thomas | Blackwell |
| John | Blangero |
| Eric | Boerwinkle |
| Donald W. | Bowden |
| Russell | Bowler |
| Jennifer | Brody |
| Ulrich | Broeckel |
| Jai | Broome |
| Deborah | Brown |
| Karen | Bunting |
| Esteban | Burchard |
| Carlos | Bustamante |
| Erin | Buth |
| Brian | Cade |
| Jonathan | Cardwell |
| Vincent | Carey |
| Julie | Carrier |
| April | Carson |
| Cara | Carty |
| Richard | Casaburi |
| Juan P Casas | Romero |
| James | Casella |
| Peter | Castaldi |
| Mark | Chaffin |
| Christy | Chang |
| Yi-Cheng | Chang |
| Daniel | Chasman |
| Sameer | Chavan |
| Bo-Juen | Chen |
| Wei-Min | Chen |
| Yii-Der Ida | Chen |
| Michael | Cho |
| Seung Hoan | Choi |
| Lee-Ming | Chuang |
| Mina | Chung |
| Ren-Hua | Chung |
| Clary | Clish |
| Suzy | Comhair |
| Matthew | Conomos |
| Elaine | Cornell |
| Adolfo | Correa |
| Carolyn | Crandall |
| James | Crapo |
| L. Adrienne | Cupples |
| Joanne | Curran |
| Jeffrey | Curtis |
| Brian | Custer |
| Coleen | Damcott |
| Dawood | Darbar |
| Sean | David |
| Colleen | Davis |
| Michelle | Daya |
| Mariza de | Andrade |
| Lisa de las | Fuentes |
| Paul de | Vries |
| Michael | DeBaun |
| Ranjan | Deka |
| Dawn | DeMeo |
| Scott | Devine |
| Huyen | Dinh |
| Harsha | Doddapaneni |
| Qing | Duan |
| Shannon | Dugan-Perez |
| Ravi | Duggirala |
| Jon Peter | Durda |
| Susan K. | Dutcher |
| Charles | Eaton |
| Lynette | Ekunwe |
| Adel El | Boueiz |
| Patrick | Ellinor |
| Leslie | Emery |
| Serpil | Erzurum |
| Charles | Farber |
| Jesse | Farek |
| Tasha | Fingerlin |
| Matthew | Flickinger |
| Myriam | Fornage |
| Nora | Franceschini |
| Chris | Frazar |
| Mao | Fu |
| Stephanie M. | Fullerton |
| Lucinda | Fulton |
| Stacey | Gabriel |
| Weiniu | Gan |
| Shanshan | Gao |
| Yan | Gao |
| Margery | Gass |
| Heather | Geiger |
| Bruce | Gelb |
| Mark | Geraci |
| Soren | Germer |
| Robert | Gerszten |
| Auyon | Ghosh |
| Richard | Gibbs |
| Chris | Gignoux |
| Mark | Gladwin |
| David | Glahn |
| Stephanie | Gogarten |
| Da-Wei | Gong |
| Harald | Goring |
| Sharon | Graw |
| Kathryn J. | Gray |
| Daniel | Grine |
| Colin | Gross |
| C. Charles | Gu |
| Yue | Guan |
| Xiuqing | Guo |
| Namrata | Gupta |
| David M. | Haas |
| Jeff | Haessler |
| Michael | Hall |
| Yi | Han |
| Patrick | Hanly |
| Daniel | Harris |
| Nicola L. | Hawley |
| Jiang | He |
| Ben | Heavner |
| Susan | Heckbert |
| Ryan | Hernandez |
| David | Herrington |
| Craig | Hersh |
| Bertha | Hidalgo |
| James | Hixson |
| Brian | Hobbs |
| John | Hokanson |
| Elliott | Hong |
| Karin | Hoth |
| Chao (Agnes) | Hsiung |
| Jianhong | Hu |
| Yi-Jen | Hung |
| Haley | Huston |
| Chii Min | Hwu |
| Marguerite Ryan | Irvin |
| Rebecca | Jackson |
| Deepti | Jain |
| Cashell | Jaquish |
| Jill | Johnsen |
| Andrew | Johnson |
| Craig | Johnson |
| Rich | Johnston |
| Kimberly | Jones |
| Hyun Min | Kang |
| Robert | Kaplan |
| Sharon | Kardia |
| Shannon | Kelly |
| Eimear | Kenny |
| Michael | Kessler |
| Alyna | Khan |
| Ziad | Khan |
| Wonji | Kim |
| John | Kimoff |
| Greg | Kinney |
| Barbara | Konkle |
| Charles | Kooperberg |
| Holly | Kramer |
| Christoph | Lange |
| Ethan | Lange |
| Leslie | Lange |
| Cathy | Laurie |
| Cecelia | Laurie |
| Meryl | LeBoff |
| Jiwon | Lee |
| Sandra | Lee |
| Wen-Jane | Lee |
| Jonathon | LeFaive |
| David | Levine |
| Dan | Levy |
| Joshua | Lewis |
| Xiaohui | Li |
| Yun | Li |
| Henry | Lin |
| Honghuang | Lin |
| Xihong | Lin |
| Simin | Liu |
| Yongmei | Liu |
| Yu | Liu |
| Ruth J.F. | Loos |
| Steven | Lubitz |
| Kathryn | Lunetta |
| James | Luo |
| Ulysses | Magalang |
| Michael | Mahaney |
| Barry | Make |
| Ani | Manichaikul |
| Alisa | Manning |
| JoAnn | Manson |
| Lisa | Martin |
| Melissa | Marton |
| Susan | Mathai |
| Rasika | Mathias |
| Susanne | May |
| Patrick | McArdle |
| Merry-Lynn | McDonald |
| Sean | McFarland |
| Stephen | McGarvey |
| Daniel | McGoldrick |
| Caitlin | McHugh |
| Becky | McNeil |
| Hao | Mei |
| James | Meigs |
| Vipin | Menon |
| Luisa | Mestroni |
| Ginger | Metcalf |
| Deborah A | Meyers |
| Emmanuel | Mignot |
| Julie | Mikulla |
| Nancy | Min |
| Mollie | Minear |
| Ryan L | Minster |
| Braxton D. | Mitchell |
| Matt | Moll |
| Zeineen | Momin |
| May E. | Montasser |
| Courtney | Montgomery |
| Donna | Muzny |
| Josyf C | Mychaleckyj |
| Girish | Nadkarni |
| Rakhi | Naik |
| Take | Naseri |
| Pradeep | Natarajan |
| Sergei | Nekhai |
| Sarah C. | Nelson |
| Bonnie | Neltner |
| Caitlin | Nessner |
| Deborah | Nickerson |
| Osuji | Nkechinyere |
| Kari | North |
| Jeff | O'Connell |
| Tim | O'Connor |
| Heather | Ochs-Balcom |
| Geoffrey | Okwuonu |
| Allan | Pack |
| David T. | Paik |
| Nicholette | Palmer |
| James | Pankow |
| George | Papanicolaou |
| Cora | Parker |
| Gina | Peloso |
| Juan Manuel | Peralta |
| Marco | Perez |
| James | Perry |
| Ulrike | Peters |
| Patricia | Peyser |
| Lawrence S | Phillips |
| Jacob | Pleiness |
| Toni | Pollin |
| Wendy | Post |
| Julia Powers | Becker |
| Meher Preethi | Boorgula |
| Michael | Preuss |
| Bruce | Psaty |
| Pankaj | Qasba |
| Dandi | Qiao |
| Zhaohui | Qin |
| Nicholas | Rafaels |
| Laura | Raffield |
| Mahitha | Rajendran |
| Vasan S. | Ramachandran |
| D.C. | Rao |
| Laura | Rasmussen-Torvik |
| Aakrosh | Ratan |
| Susan | Redline |
| Robert | Reed |
| Catherine | Reeves |
| Elizabeth | Regan |
| Alex | Reiner |
| Muagututi |  |
| a Sefuiva | Reupena |
| Ken | Rice |
| Stephen | Rich |
| Rebecca | Robillard |
| Nicolas | Robine |
| Dan | Roden |
| Carolina | Roselli |
| Jerome | Rotter |
| Ingo | Ruczinski |
| Alexi | Runnels |
| Pamela | Russell |
| Sarah | Ruuska |
| Kathleen | Ryan |
| Ester Cerdeira | Sabino |
| Danish | Saleheen |
| Shabnam | Salimi |
| Sejal | Salvi |
| Steven | Salzberg |
| Kevin | Sandow |
| Vijay G. | Sankaran |
| Jireh | Santibanez |
| Karen | Schwander |
| David | Schwartz |
| Frank | Sciurba |
| Christine | Seidman |
| Jonathan | Seidman |
| Frédéric | Sériès |
| Vivien | Sheehan |
| Stephanie L. | Sherman |
| Amol | Shetty |
| Aniket | Shetty |
| Wayne Hui- Heng | Sheu |
| M. Benjamin | Shoemaker |
| Brian | Silver |
| Edwin | Silverman |
| Robert | Skomro |
| Albert Vernon | Smith |
| Jennifer | Smith |
| Josh | Smith |
| Nicholas | Smith |
| Tanja | Smith |
| Sylvia | Smoller |
| Beverly | Snively |
| Michael | Snyder |
| Tamar | Sofer |
| Nona | Sotoodehnia |
| Adrienne M. | Stilp |
| Garrett | Storm |
| Elizabeth | Streeten |
| Jessica Lasky | Su |
| Yun Ju | Sung |
| Jody | Sylvia |
| Adam | Szpiro |
| Daniel | Taliun |
| Hua | Tang |
| Margaret | Taub |
| Kent D. | Taylor |
| Matthew | Taylor |
| Simeon | Taylor |
| Marilyn | Telen |
| Timothy A. | Thornton |
| Machiko | Threlkeld |
| Lesley | Tinker |
| David | Tirschwell |
| Sarah | Tishkoff |
| Hemant | Tiwari |
| Catherine | Tong |
| Russell | Tracy |
| Michael | Tsai |
| Dhananjay | Vaidya |
| David Van Den | Berg |
| Peter | VandeHaar |
| Scott | Vrieze |
| Tarik | Walker |
| Robert | Wallace |
| Avram | Walts |
| Fei Fei | Wang |
| Heming | Wang |
| Jiongming | Wang |
| Karol | Watson |
| Jennifer | Watt |
| Daniel E. | Weeks |
| Joshua | Weinstock |
| Bruce | Weir |
| Scott T | Weiss |
| Lu-Chen | Weng |
| Jennifer | Wessel |
| Cristen | Willer |
| Kayleen | Williams |
| L. Keoki | Williams |
| Carla | Wilson |
| James | Wilson |
| Lara | Winterkorn |
| Quenna | Wong |
| Joseph | Wu |
| Huichun | Xu |
| Lisa | Yanek |
| Ivana | Yang |
| Ketian | Yu |
| Seyedeh | Maryam Zekavat |
| Yingze | Zhang |
| Snow Xueyan | Zhao |
| Wei | Zhao |
| Xiaofeng | Zhu |
| Michael | Zody |
| Sebastian | Zoellner |
