# Supplementary material for: Polygenic scores for obstructive sleep apnoea reveal pathways contributing to cardiovascular disease
Source: eBioMedicine. 2025 Jun 4;117:105790. doi: 10.1016/j.ebiom.2025.105790 (PMC12171572; doi:10.1016/j.ebiom.2025.105790)
Supplement: Supplementary File 3 [file mmc3.docx]

| **First names** | **Surnames** |
| --- | --- |
| Aarno | Palotie |
| Mark | Daly |
| Bridget | Riley-Gills |
| Howard | Jacob |
| Coralie | Viollet |
| Slavé | Petrovski |
| Chia-Yen | Chen |
| Sally | John |
| George | Okafo |
| Robert | Plenge |
| Joseph | Maranville |
| Mark | McCarthy |
| Rion | Pendergrass |
| Jonathan | Davitte |
| Kirsi | Auro |
| Simonne | Longerich |
| Anders | Mälarstig |
| Anna | Vlahiotis |
| Katherine | Klinger |
| Clement | Chatelain |
| Jorg | Blankenstein |
| Karol | Estrada |
| Robert | Graham |
| Dawn | Waterworth |
| Chris | OÂ´Donnell |
| Nicole | Renaud |
| Tomi P | P. Mäkelä |
| Jaakko | Kaprio |
| Minna | Ruddock |
| Petri | Virolainen |
| Antti | Hakanen |
| Terhi | Kilpi |
| Markus | Perola |
| Jukka | Partanen |
| Taneli | Raivio |
| Jani | Tikkanen |
| Raisa | Serpi |
| Kati | Kristiansson |
| Veli-Matti | Kosma |
| Jari | Laukkanen |
| Marco | Hautalahti |
| Outi | Tuovila |
| Jeffrey | Waring |
| Bridget | Riley-Gillis |
| Fedik | Rahimov |
| Ioanna | Tachmazidou |
| Zhihao | Ding |
| Marc | Jung |
| Hanati | Tuoken |
| Shameek | Biswas |
| Neha | Raghavan |
| Adriana | Huertas-Vazquez |
| Jae-Hoon | Sul |
| Xinli | Hu |
| Åsa | Hedman |
| Ma´en | Obeidat |
| Jonathan | Chung |
| Jonas | Zierer |
| Mari | Niemi |
| Samuli | Ripatti |
| Johanna | Schleutker |
| Mikko | Arvas |
| Olli | Carpen |
| Reetta | Hinttala |
| Johannes | Kettunen |
| Arto | Mannermaa |
| Katriina Aalto- | Setälä |
| Mika | Kähönen |
| Johanna | Mäkelä |
| Reetta | Kälviäinen |
| Valtteri | Julkunen |
| Hilkka | Soininen |
| Anne | Remes |
| Mikko | Hiltunen |
| Jukka | Peltola |
| Minna | Raivio |
| Pentti | Tienari |
| Juha | Rinne |
| Roosa | Kallionpää |
| Juulia | Partanen |
| Adam | Ziemann |
| Nizar | Smaoui |
| Anne | Lehtonen |
| Susan | Eaton |
| Heiko | Runz |
| Sanni | Lahdenperä |
| Natalie | Bowers |
| Edmond | Teng |
| Fanli | Xu |
| David | Pulford |
| Laura | Addis |
| John | Eicher |
| Qingqin S | Li |
| Karen | He |
| Ekaterina | Khramtsova |
| Martti | Färkkilä |
| Jukka | Koskela |
| Sampsa | Pikkarainen |
| Airi | Jussila |
| Katri | Kaukinen |
| Timo | Blomster |
| Mikko | Kiviniemi |
| Markku | Voutilainen |
| Tim | Lu |
| Linda | McCarthy |
| Amy | Hart |
| Meijian | Guan |
| Jason | Miller |
| Kirsi | Kalpala |
| Melissa | Miller |
| Kari | Eklund |
| Antti | Palomäki |
| Pia | Isomäki |
| Laura | Pirilä |
| Oili | Kaipiainen-Seppänen |
| Johanna | Huhtakangas |
| Nina | Mars |
| Apinya | Lertratanakul |
| Marla | Hochfeld |
| Jorge Esparza | Gordillo |
| Fabiana | Farias |
| Nan | Bing |
| Tarja | Laitinen |
| Margit | Pelkonen |
| Paula | Kauppi |
| Hannu | Kankaanranta |
| Terttu | Harju |
| Riitta | Lahesmaa |
| Hubert | Chen |
| Joanna | Betts |
| Rajashree | Mishra |
| Majd | Mouded |
| Debby | Ngo |
| Teemu | Niiranen |
| Felix | Vaura |
| Veikko | Salomaa |
| Kaj | Metsärinne |
| Jenni | Aittokallio |
| Jussi | Hernesniemi |
| Daniel | Gordin |
| Juha | Sinisalo |
| Marja-Riitta | Taskinen |
| Tiinamaija | Tuomi |
| Timo | Hiltunen |
| Amanda | Elliott |
| Mary Pat | Reeve |
| Sanni | Ruotsalainen |
| Dirk | Paul |
| Audrey | Chu |
| Dermot | Reilly |
| Mike | Mendelson |
| Jaakko | Parkkinen |
| Tuomo | Meretoja |
| Heikki | Joensuu |
| Johanna | Mattson |
| Eveliina | Salminen |
| Annika | Auranen |
| Peeter | Karihtala |
| Päivi | Auvinen |
| Klaus | Elenius |
| Esa | PitkÃ¤nen |
| Relja | Popovic |
| Margarete | Fabre |
| Jennifer | Schutzman |
| Diptee | Kulkarni |
| Alessandro | Porello |
| Andrey | Loboda |
| Heli | Lehtonen |
| Stefan | McDonough |
| Sauli | Vuoti |
| Kai | Kaarniranta |
| Joni A | Turunen |
| Terhi | Ollila |
| Hannu | Uusitalo |
| Juha | Karjalainen |
| Mengzhen | Liu |
| Stephanie | Loomis |
| Erich | Strauss |
| Hao | Chen |
| Kaisa | Tasanen |
| Laura | Huilaja |
| Katariina | Hannula-Jouppi |
| Teea | Salmi |
| Sirkku | Peltonen |
| Leena | Koulu |
| David | Choy |
| Ying | Wu |
| Pirkko | Pussinen |
| Aino | Salminen |
| Tuula | Salo |
| David | Rice |
| Pekka | Nieminen |
| Ulla | Palotie |
| Maria | Siponen |
| Liisa | Suominen |
| Päivi | Mäntylä |
| Ulvi | Gursoy |
| Vuokko | Anttonen |
| Kirsi | Sipilä |
| Hannele | Laivuori |
| Venla | Kurra |
| Laura | Kotaniemi-Talonen |
| Oskari | Heikinheimo |
| Ilkka | Kalliala |
| Lauri | Aaltonen |
| Varpu | Jokimaa |
| Marja | Vääräsmäki |
| Outi | Uimari |
| Laure | Morin-Papunen |
| Maarit | Maarit |
| Terhi | Piltonen |
| Katja | Kivinen |
| Elisabeth | Widen |
| Taru | Tukiainen |
| Niko | Välimäki |
| Eija | Laakkonen |
| Jaakko | Tyrmi |
| Heidi | Silven |
| Eeva | Sliz |
| Riikka | Arffman |
| Susanna | Savukoski |
| Triin | Laisk |
| Natalia | Pujol |
| Janet | Kumar |
| Iiris | Hovatta |
| Erkki | Isometsä |
| Hanna | Ollila |
| Jaana | Suvisaari |
| Antti | Mäkitie |
| Argyro | Bizaki-Vallaskangas |
| Sanna | Toppila-Salmi |
| Tytti | Willberg |
| Elmo | Saarentaus |
| Antti | Aarnisalo |
| Elisa | Rahikkala |
| Kristiina | Aittomäki |
| Fredrik | Åberg |
| Mitja | Kurki |
| Aki | Havulinna |
| Juha | Mehtonen |
| Priit | Palta |
| Shabbeer | Hassan |
| Pietro Della Briotta | Parolo |
| Wei | Zhou |
| Mutaamba | Maasha |
| Susanna | Lemmelä |
| Manuel | Rivas |
| Aoxing | Liu |
| Arto | Lehisto |
| Andrea | Ganna |
| Vincent | Llorens |
| Henrike | Heyne |
| Joel | Rämö |
| Satu | Strausz |
| Tuula | Palotie |
| Kimmo | Palin |
| Javier | Garcia-Tabuenca |
| Harri | Siirtola |
| Tuomo | Kiiskinen |
| Jiwoo | Lee |
| Kristin | Tsuo |
| Kati | Hyva ̈rinen |
| Jarmo | Ritari |
| Katri | Pylkäs |
| Minna | Karjalainen |
| Tuomo | Mantere |
| Eeva | Kangasniemi |
| Sami | Heikkinen |
| Nina | Pitkänen |
| Samuel | Lessard |
| Clément | Chatelain |
| Lila | Kallio |
| Tiina | Wahlfors |
| Eero | Punkka |
| Sanna | Siltanen |
| Tiina | Jokela |
| Anu | Jalanko |
| Auli | Toivola |
| Huei-Yi | Shen |
| Risto | Kajanne |
| Rodos | Rodosthenous |
| Mervi | Aavikko |
| Helen | Cooper |
| Denise | Öller |
| Rasko | Leinonen |
| Henna | Palin |
| Malla-Maria | Linna |
| Masahiro | Kanai |
| Zhili | Zheng |
| L. Elisa | Lahtela |
| Mari | Kaunisto |
| Elina | Kilpeläinen |
| Tianduanyi | Wang |
| Timo | P. Sipilä |
| Oluwaseun | Alexander Dada |
| Awaisa | Ghazal |
| Anastasia | Kytölä |
| Rigbe | Weldatsadik |
| Jaska | Uimonen |
| Kati | Donner |
| Anu | Loukola |
| Päivi | Laiho |
| Tuuli | Sistonen |
| Essi | Kaiharju |
| Markku | Laukkanen |
| Elina | Järvensivu |
| Sini | Lähteenmäki |
| Lotta | Männikkö |
| Regis | Wong |
| Minna | Brunfeldt |
| Sami | Koskelainen |
| Tero | Hiekkalinna |
| Teemu | Paajanen |
| Shuang | Luo |
| Shanmukha | Sampath Padmanabhuni |
| Marianna | Niemi |
| Javier | Gracia-Tabuenca |
| Mika | Helminen |
| Tiina | Luukkaala |
| Iida | Vähätalo |
| Iina | Laak |
| Saija | Haapa-Paananen |
| Sarah | Smith |
| Tom | Southerington |
| Meri | Lähteenmäki |
